# National Policies to Limit Food Marketing and Competitive Food Sales in Schools: A Global Scoping Review

**DOI:** 10.1016/j.advnut.2024.100254

**Published:** 2024-06-12

**Authors:** Michelle Perry, Kayla Mardin, Grace Chamberlin, Emily A Busey, Lindsey Smith Taillie, Francesca R Dillman Carpentier, Barry M Popkin

**Affiliations:** 1 Global Food Research Program, Carolina Population Center, University of North Carolina at Chapel Hill, Chapel Hill, NC, United States; 2 Department of Nutrition, Gillings School of Global Public Health, University of North Carolina at Chapel Hill, Chapel Hill, NC, United States; 3 Department of Health Policy, Gillings School of Global Public Health, University of North Carolina at Chapel Hill, Chapel Hill, NC, United States; 4 Hussman School of Journalism and Media, University of North Carolina at Chapel Hill, Chapel Hill, NC, United States

**Keywords:** school food environment, food policy, food marketing, ultraprocessed foods, childhood obesity, scoping review

## Abstract

School food environments contribute to children’s nutritional intake and overall health. As such, the World Health Organization and other public health organizations encourage policies that restrict children’s access and exposure to foods and beverages that do not build health in and around schools. This global scoping review explores the presence and characteristics of policies that restrict competitive food sales and marketing for unhealthy foods across 193 countries using evidence from policy databases, gray literature, peer-reviewed literature, and primary policy documents. Policies were included if they were nationally mandated and regulated marketing and/or competitive foods in the school environments. Worldwide, only 28% of countries were found to have any national-level policy restricting food marketing or competitive food sales in schools: 16% of countries restrict marketing, 25% restrict competitive foods, and 12% restrict both. Over half of policies were found in high-income countries. No low-income countries had either policy type. Eight marketing policies (27%) and 14 competitive foods policies (29%) lacked explicit guidelines for either policy monitoring or enforcement. Future research is needed to assess the prevalence of policies aimed at improving other key aspects of the school food environment, such as dietary quality of school meals or food procurement, as well as assess the implementation and efficacy of existing policies.

**Statement of Significance**

This article presents the first scoping review of national policies restricting competitive food sales and marketing in the school food environment in every country, worldwide.

## Introduction

The school food environment has long been recognized as a key contributor to children’s nutritional intake and overall health [1]. In many countries, children consume up to a third of their daily energy intake at school [[1], [2], [3]]. As children’s diets worldwide have shifted toward energy-dense, ultraprocessed foods and drinks high in sugar, salt, and saturated fat and often devoid of essential nutrients [1], policies to promote healthier school food environments offer a promising way to improve children’s health by providing essential nutrients and establishing healthy eating habits early in life.

Improving the school food environment is of particular interest to policymakers worldwide, given global increases in child and adolescent overweight and obesity and earlier onset of other nutrition-related risk factors and diseases such as type 2 diabetes, hypertension, and liver disease [[4], [5], [6], [7], [8], [9]]. An estimated 390 million children ages 5–19 y around the world were classified as overweight or obese in 2022—an increase from roughly 1 in 12 children in 1990 to 1 in 5 today [10,11]—and prevalence among preschoolers has risen over 60% since 1990 [12]. Well-designed school feeding programs and school food policies can ideally limit overnutrition while also mitigating undernutrition, particularly in places where stunting and micronutrient deficiencies are prevalent.

The WHO has offered guidance for developing regulatory interventions to influence the school food environment, which encompasses all the spaces and conditions inside and around schools where food is available, obtained, or consumed [13,14]. Among these recommendations, the WHO suggests setting nutrition standards for foods served and sold in the school environment and restricting the marketing of unhealthy foods and beverages in this environment [13]. Yet, little documentation exists as to the nature of such policies on a global scale or evidence on the implementation and impact of these policies [[15], [16], [17], [18], [19], [20], [21], [22], [23]].

This scoping review describes the global prevalence and policy features of 2 key school food policy types: *1*) restrictions on competitive foods sales outside of school meals, and *2*) restrictions on food and beverage marketing in and around schools. More specifically, this review captures mandatory policies implemented at the national-level. A companion review describing national policies to limit nutrients, ingredients, or categories of concern in school meals is forthcoming and will be published separately.

## Methods

Researchers conducted a scoping review to identify and describe national-level, mandatory school food environment policies regulating competitive food sales and/or food marketing in all countries in the world. The protocol for this review, including the research question, data sources and search strategy, process for selection of evidence and data coding, and synthesis, was determined in advance and registered in the Open Science Framework [24]. The review was conducted in accordance with the PRISMA Extension for Scoping Reviews checklist and using search strategies described in previous literature on applying systematic search methods to gray literature [25,26]. Key terms are defined in Table 1.

**TABLE 1:** Key terms

Term	Definition
Policy	Any legislation, regulation, standard, or guideline that meets the inclusion criteria laid out in this search
National policy	Policies adopted by a national government that apply to the entire country
Mandatory policy	Policies that are statutory or mandated by law. This excludes nonbinding recommendations and suggested guidelines (for example, general national dietary guidelines are not included unless a national policy requires adherence to them in school settings)
Schools	Educational institutions offer learning for students under the instruction of teachers through graduating secondary/high school
Complete restriction	A full ban on either competitive food sales (that is, no foods or beverages may be sold outside of school meals at schools) or marketing (that is, no food/beverage marketing of any kind is allowed on and/or around school grounds)
Partial restriction	A policy that restricts competitive food/beverage sales or marketing for certain foods and beverages but does not completely prohibit either action
Categorical restriction	Limit on competitive food sales or marketing applied to a list of specific food types or categories (for example, chips and sweets), regardless of the individual food or beverage items’ nutritional profile
Nutrient restriction	Limit on competitive food/beverage sales or marketing applied to foods/beverages based on their nutritional content (for example, grams of sodium or total calories per specified unit); nutrients in this review include calories, total sugar, added sugar, total fat, saturated fat, trans fat, and sodium
Ingredient restriction	Limit on competitive food/beverage sales or marketing applied to foods/beverages containing a specific ingredient. Ingredients in this review include nonnutritive sweeteners and caffeine
Competitive foods	Any foods or beverages sold in schools outside of a national school meal program. This includes foods sold in canteens, kiosks, tuck shops, vending machines, and from vendors coming onto school grounds
Food marketing	Any oral, written, or graphic statements made to promote the sale of a food or beverage product
Surrounding area	Any area around school grounds. Policies coded as applying to the surrounding area must specify the surrounding area and/or the distance to which the restrictions reach (for example, a 200-meter radius)

### Sample

All 193 countries designated as United Nations member countries as of September 12, 2022, were included in this review (Appendix 1) [27]. Analysis of trends by income level and world region used the World Bank’s fiscal year 2023 income and region classifications [28].

### Search and data sources

To identify active policies and primary regulatory documents, an iterative search process was conducted using the following sources: *1*) existing global policy databases [for example, The World Cancer Research Fund’s NOURISHING Database, The WHO’s Global database on the Implementation of Nutrition Action (GINA) [29], and the Global Obesity Observatory]; *2*) peer-reviewed literature; *3*) official government websites; *4*) Internet search engines; and *5*) in-country contacts for clarifying ambiguity in policy interpretation or identification. These contacts include staff from UNICEF and the World Food Programme, national ministries related to food, health, and/or education, and faculty from research universities. We found in-country contacts either through government websites, authors of papers related to school food in that country, or through personal academic connections. Peer-reviewed articles and gray literature were used to identify and validate the existence or absence of policies and their evolution over time if policies were not immediately located through a search.

For each country, researchers identified any policies possibly meeting inclusion criteria in existing global policy databases. When a relevant policy was found, primary policy documentation was sought either via the database, internet search engine, or other sources listed above. If a country had no policy listed in global databases, researchers then searched gray and peer-reviewed literature to identify the existence of a policy and locate primary policy documentation, when applicable. These steps were repeated for each country (Supplemental Figure 1).

When using search engines to identify policies, search terms for each country included the country name and combinations of the following search terms: “school,” “cafeteria,” “canteen,” “food,” “beverage,” “nutrition,” “policy,” “regulation,” “restriction,” “guidelines,” “competitive foods,” “sale,” “sell,” “marketing,” and “advertising.”

No limits were placed on sources’ language or publication date to capture as much information as possible. Due to limited resources, Google Translate was used to translate any policies into English [30]. For policies that had publicly available English versions, these versions were used for coding. Any translated policy language was cross-referenced with policy language included in other databases like GINA, when available. If any concerns arose about the quality of the policy translation, the next steps included seeking out native speakers, either through reaching out to nutrition policy experts in the country or other personal or research connections.

The following methods were utilized to translate and interpret policies written in languages other than English: *1*) Google Translate: Documents were translated using the Google Translate text, website, and document functions; *2*) Translators: Native speakers were utilized to translate and interpret documents when available; and *3*) Corroborated information: researchers compared the translated policies to information found in other review sources (for example, from policy databases). Ultimately, 19 policy documents required further clarification after the initial translation process. When doubt remained about the accuracy of translation or interpretation of policy wording/meaning after completion of these 3 steps, researchers reached out to researchers or ministry officials in the country of interest for clarification. After these steps, any unresolved questions were discussed with the research team and, after consensus was reached, the texts were coded using the information available. Countries requiring additional clarification from in-country experts are noted in Appendix 1. The search was conducted between September 2022 and June 2023. Policies were coded as active as of the date of data collection and coding. All coded information was ultimately extracted from a primary, national policy document.

### Policy inclusion/exclusion criteria

Researchers identified active, national-level, mandatory policies that included either restrictions on competitive food sales (that is, policies that limited access to, use of, or sale of certain products) or food marketing in and around schools. Restriction is defined as a limit or ban on commercial practices (sales or marketing) related to foods and beverages in or around schools; language must indicate clear restriction (for example, “prohibited,” or “may not be sold”). Mandatory policies are defined as those that are statutory or mandated by law in the country where they are implemented. This excludes nonbinding recommendations and suggested guidelines.

Policies implemented by sub-national jurisdictions (for example, states, territories, provinces, or cities) or not containing restrictive language were excluded from this review. As outlined in the registered review protocol [24], data were also gathered on policies setting certain nutritional standards for school meals, but this will be analyzed and reported in a future article.

Schools are defined as educational institutions offering learning for students under the instruction of teachers through graduating secondary/high school. This review excludes colleges and universities, after-school programs, and “social care” or “childcare” institutions. Grade levels and ages were standardized into the following categories because of an inconsistency in use across policies: *1*) Preschools: children under 6 years of age, prekindergarten; *2*) Primary schools: grades kindergarten–5 or children between 6 and 11 y of age; *3*) Lower-secondary schools: grades 6–8 or children between 11 and 13 y of age; and *4*) Upper-secondary schools: grades 9–12 or children between 13 and 18 y of age.

### Data collection

A codebook was developed in collaboration with researchers with expertise in marketing regulations and school food policies (Appendix 2). The codebook was reviewed and piloted by the research team and modified to meet research objectives and ensure researchers’ responses were consistent across questions. Data extracted included the existence of marketing or competitive food restrictions, target grade/age, specific category and nutrient criteria, and monitoring and enforcement language. All data collection occurred between August 2022 and June 2023. Changes to policies or new policies introduced after this timeframe are not included in this dataset.

Researchers used REDCap (Research Electronic Data Capture) for data collection [31,32]. The REDCap Double Data Entry module was used to assess inter-rater reliability for a random sample of 20 countries. Any discrepancies were reviewed as a group and consensus-coded. The 2 coders had 85% agreement across all codebook variables, indicating sufficient inter-rater reliability. Researchers then independently coded the remaining 173 countries and raised any questions for further review with the research team.

## Results

### Global overview

Worldwide, 28% of countries (*n* = 54) were found to have any national-level policy restricting food marketing and/or competitive food sales in schools: 16% of countries (*n* = 30) include marketing restrictions (Table 2) and 25% (*n* = 48) include restrictions on competitive foods in schools (Table 3). (See policy descriptions in Supplemental Table 1) Six countries had marketing restrictions only; 24 had competitive food restrictions only, and 12% of all countries (*n* = 24) restricted both marketing and competitive food sales. Among the 54 countries with regulations, restrictions on competitive food sales were much more common (89% of policies) than limits on marketing for unhealthy foods and beverages (56% of policies).

**TABLE 2:**
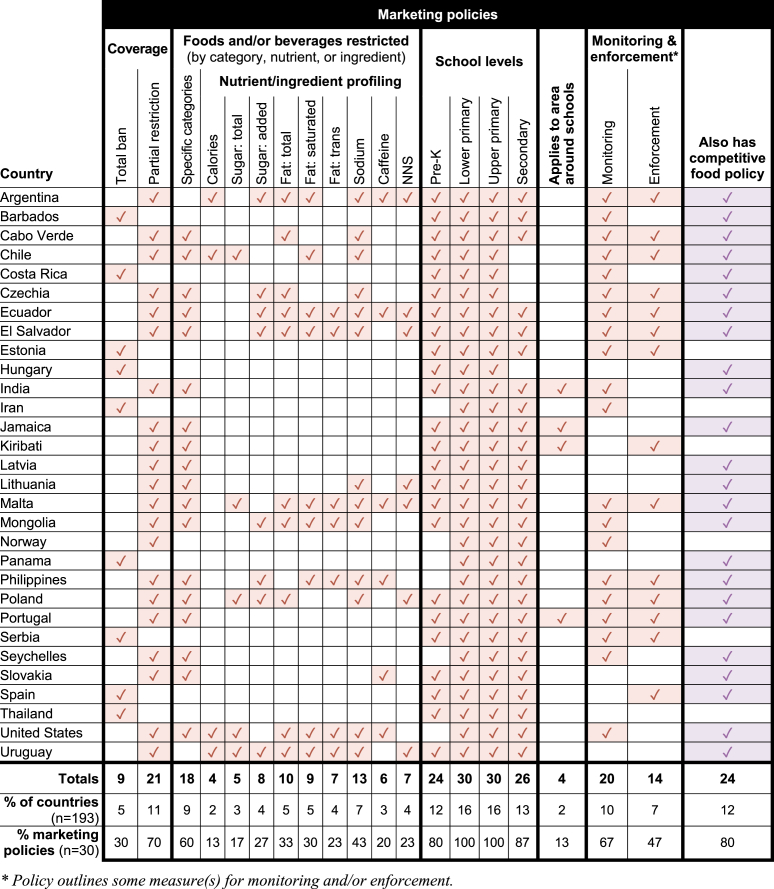
Countries with national policies regulating food marketing in or around schools

**TABLE 3:**
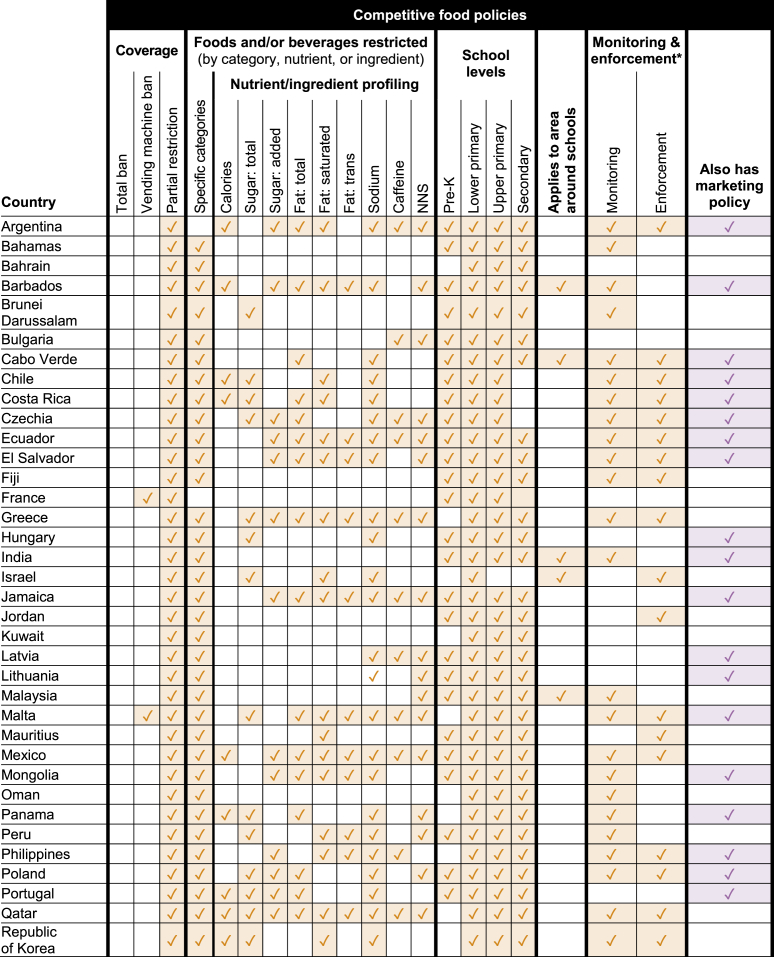

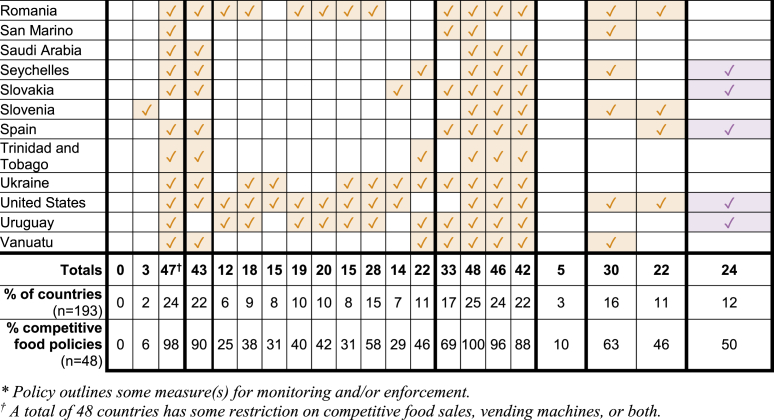
Countries with national policies restricting competitive food sales in schools

Among all policies, restrictions most often applied to primary schools (100% of policies), followed closely by middle schools (96%), then high schools (89%), and finally preschools (70%). The majority of policies applied to all age categories or all public academic institutions, with preschools most often omitted from policy language. This pattern was consistent across both policy areas: Among marketing policies, 100% applied to primary and middle schools, while fewer applied to high schools (87%) and preschools (80%). Similarly, 100% of competitive food restrictions applied to primary schools, 96% to middle schools, 88% to high schools, and 69% to preschools. Marketing restrictions more commonly applied to all 4 school age groups.

### Marketing policies

**FIGURE 1:**
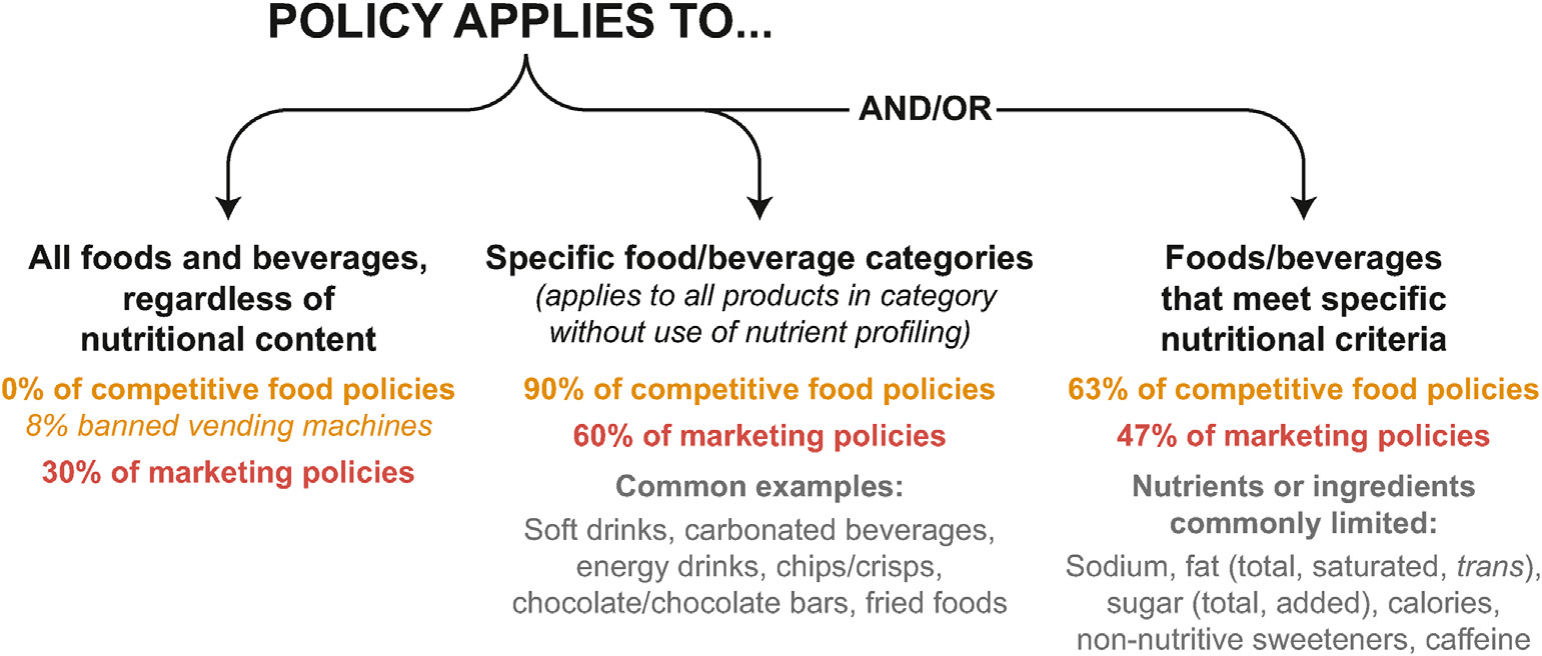
Policy approaches to determining which foods and beverages cannot be sold as competitive foods or marketed in schools.

Of the 30 countries with policies restricting food marketing, 9 policies (30%) explicitly banned any form of marketing or any food and beverage marketing on school grounds. The remaining 21 policies (70%) listed specific categories or outlined nutritional criteria for determining which food and beverage products cannot be marketed (Figure 1).

Most policies limited marketing for specific categories of foods (*n* = 18, 60%) and/or used nutritional or ingredient criteria (*n* = 14, 47%), based on an established nutrient profiling model (for example, Pan American Health Organization Nutrient Profile Model [33]), national dietary guidelines, or thresholds set specifically for the policy. The most common categories subject to marketing restrictions were energy drinks (*n* = 9), soft drinks/sodas/carbonated beverages (*n* = 5), beverages that contain artificial sweeteners (*n* = 5), chocolate/chocolate bars (*n* = 4), chips (*n* = 4), and fried foods (*n* = 3). One policy (in El Salvador) restricted marketing for “ultraprocessed” foods as a category. Two countries provided lists of foods approved for in-school marketing, indicating that all other foods that are not explicitly approved are prohibited.

**FIGURE 2:**
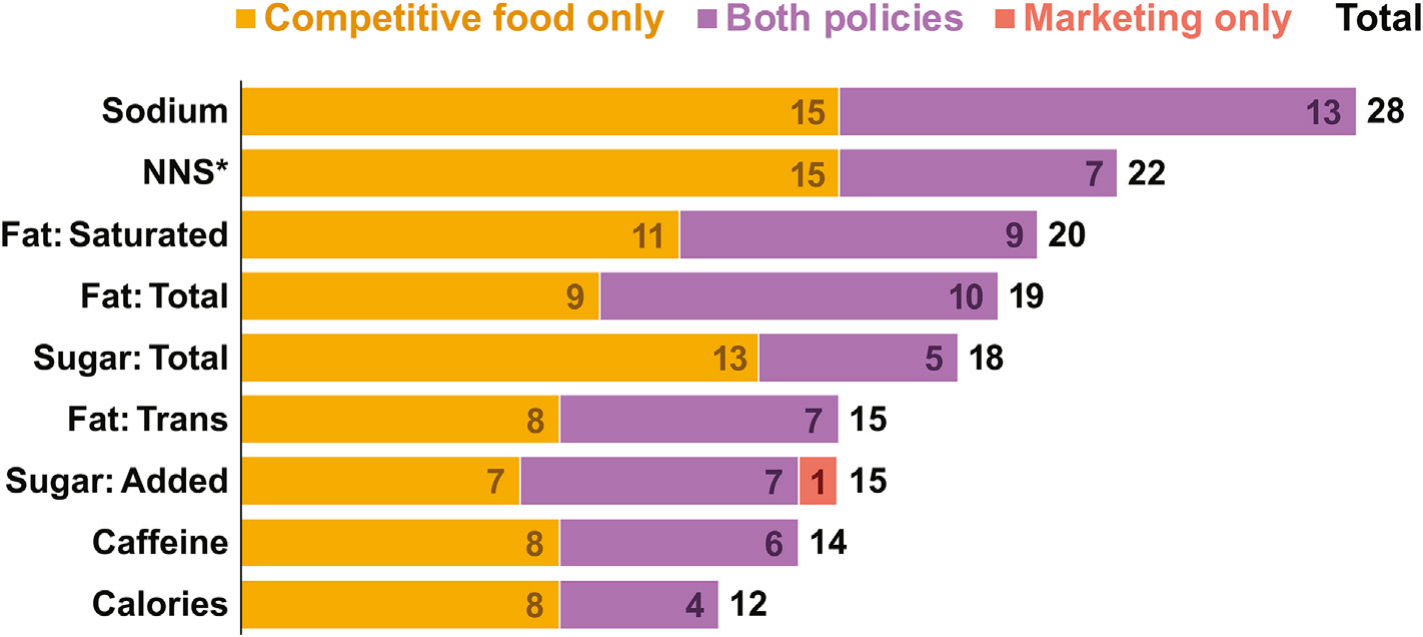
Frequency of nutrients or ingredients profiled by policies to identify foods or drinks subject to marketing restrictions and/or limited competitive food sales. NNS: Nonnutritive sweeteners.

For policies that use nutrient or ingredient content thresholds (*n* = 14), sodium was the most commonly regulated nutrient or ingredient (93%, *n* = 13), followed by total fat (71%, *n* = 10), saturated fat (64%, *n* = 9), and added sugar (57%, *n* = 8) (Figure 2). Total calories were least commonly restricted among marketing policies (29%, *n* = 4). Uruguay limited the most nutrients/ingredients in its marketing policy (8 of 9). Seven policies regulate nonnutritive sweeteners, and 6 policies regulate caffeine. See Table 2 for which countries restrict 1 or more nutrients or ingredients listed here.

All 9 total marketing bans applied to primary and lower-secondary schools, and 7 bans also covered preschools and upper-secondary schools. Policies limiting marketing for specific product categories or foods high in nutrients or ingredients of concern more often included primary and lower-secondary schools (*n* = 21) than upper-secondary schools (*n* = 19) and preschools (*n* = 17). Only 4 (13%) of marketing policies extended to the area surrounding schools.

### Competitive food policies

Forty-eight countries had a national policy restricting sales of competitive foods within schools. Only 5 (10%) of these policies extended to the area around schools, including those in Barbados, Cabo Verde, India, Jamaica, and Malaysia. No country had a total ban on competitive food sales. Forty-five policies (94%) had criteria (categorical, nutritional, or both) to determine which competitive foods could be sold on school grounds. Only 3 policies (6%) banned vending machines entirely.

Forty-three (90%) competitive food policies limited sales of specific food or beverage categories, most often explicitly banning soft drinks/sodas/fizzy drinks/carbonated beverages (*n* = 30), energy drinks (*n* = 21), chips/crisps (*n* = 18), chocolate/chocolate bars (*n* = 18), and fried foods (*n* = 14) (Figure 1). Although only 1 country explicitly regulated ultraprocessed foods (El Salvador), 4 limited or banned “processed foods” such as French fries, burgers, or sausages.

Thirty (63%) of competitive food policies utilized nutrient or ingredient thresholds or nutrient profiling models for restrictions. As with marketing policies, sodium was the most commonly restricted nutrient (93%, *n* = 28), followed by saturated fat (67%, *n* = 20), total fat (63%, *n* = 19), and total sugar (60%, *n* = 18) (Figure 2). In addition, 47% (*n* = 14) of competitive food policies with nutrient or ingredient thresholds regulated caffeine, and 73% (*n* = 22) regulated nonnutritive sweetener content in competitive food or beverages. Thirty-four countries had a policy that limited the sale of competitive foods based on both categorial and nutrient or ingredient restrictions. See Table 3 for details on nutrient restriction by country.

The grade levels most targeted by competitive food restrictions were primary schools (100%) and lower-secondary grades (96%). Eighty-eight percent (*n* = 43) of policies targeted upper-secondary grades and 69% (*n* = 34) targeted preschools.

### Monitoring and enforcement

**FIGURE 3:**
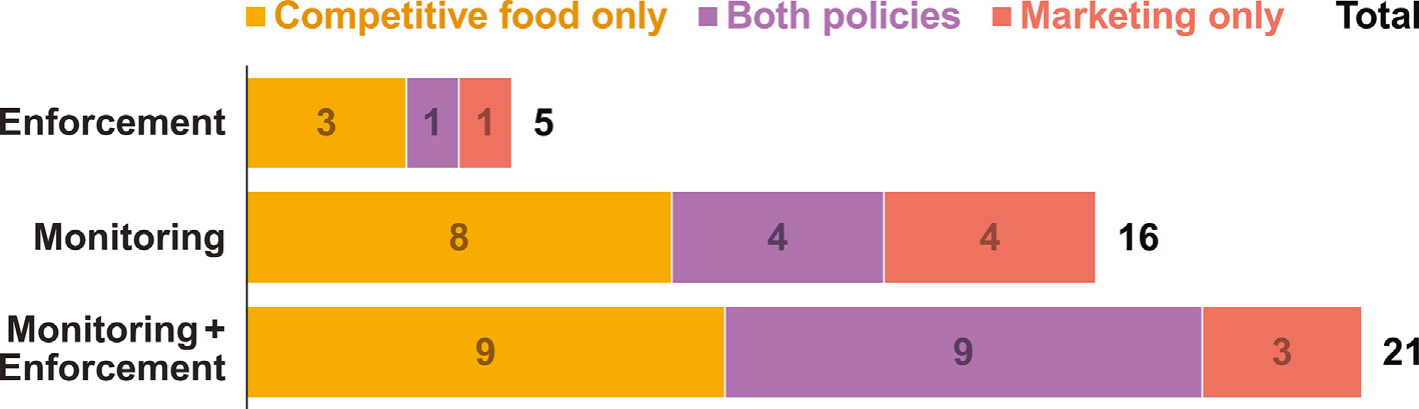
Frequency of inclusion of monitoring and enforcement plans in school marketing and competitive food policies.

Roughly two-thirds of policies—67% of marketing policies (*n* = 20) and 63% of competitive food policies (*n* = 30)—included language outlining how compliance would be monitored, most often in the form of inspection by government ministries or newly created committees associated with school feeding. In contrast, fewer than half of policies—47% (*n* = 14) of marketing policies and 46% (*n* = 22) of competitive food policies—included language outlining policy enforcement, most often detailing the use of fines or financial penalties for noncompliance. Of the 24 countries that had both a marketing and competitive food policy, 15 were found to have monitoring language and 10 were found to have enforcement language for both policies. Details for both monitoring and enforcement were outlined in 12 of the 30 marketing policies and 18 of the 48 competitive food policies (Figure 3).

### Policies by country income classification and geographic region

#### Income classification

**FIGURE 4:**
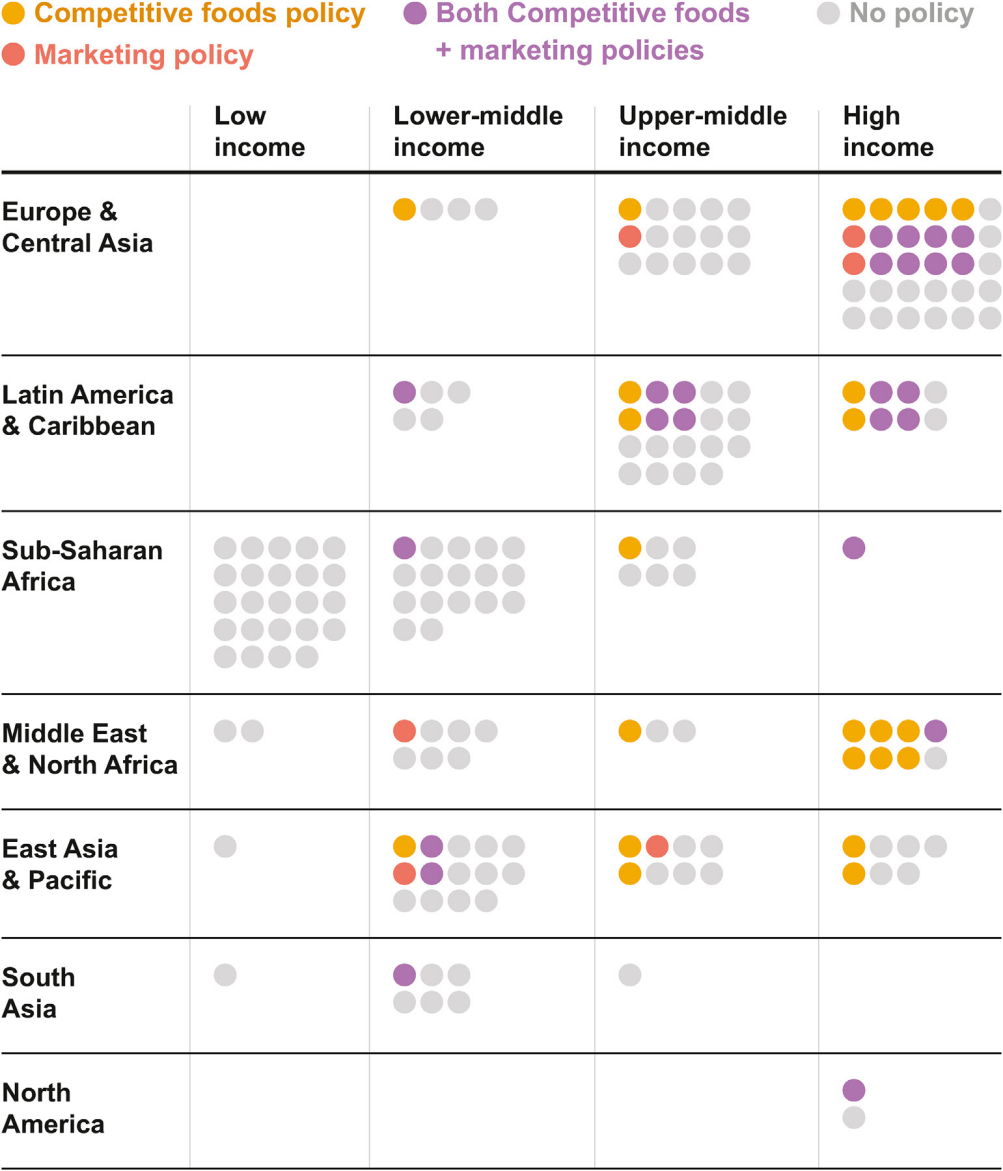
Countries with any national policy restricting in-school competitive food sales and/or marketing for unhealthy foods or drinks, by world region and income level Country income levels and world regions were determined using the World Bank’s fiscal year 2023 income and region classifications [28], which are listed in Appendix 1.

Policies were found more frequently among high-income countries (HICs) and decreased in prevalence with each income level (Figure 4 [28]). Fifty-nine percent (*n* = 32) of policies were found in HICs, 24% (*n* = 13) in upper-middle-income countries (UMICs), and 17% (*n* = 9) in lower-middle-income countries (LMICs). Eight of the 9 countries with policies outright prohibiting all marketing of foods or beverages within the school environment were HICs or UMICs. All 3 vending machine bans were found in HICs. HICs also had a higher frequency of combined competitive food and marketing policies (*n* = 15) compared with LMICs (*n* = 5) and UMICs (*n* = 4). No low-income countries (LICs) had a policy regulating either the marketing of unhealthy foods or the sale of competitive foods in the school environment.

LMICs more often included language for monitoring compliance compared with HICs and UMICs. Eighty-six percent of marketing policies in LMICs included language for monitoring compared with only 59% in HICs and 67% in UMICs. Similarly, 86% of competitive food policies in LMICs included language for monitoring compared with only 57% in HICs and 64% in UMICs. There were no notable differences regarding enforcement language by income classification.

### Geographic region

Europe & Central Asia and Latin America & Caribbean regions had the most countries with either a competitive food or marketing policy (Figure 4). Sub-Saharan Africa, South Asia, and North America had fewest countries with either policy. The Latin America & Caribbean region notably had the highest relative concentration of policies that addressed both marketing and competitive food sales. Of competitive food policies, policies in the Middle East & North Africa and East Asia & The Pacific regions were more likely to have categorical bans than nutrient bans.

## Discussion

This scoping review found that most countries did not have national, mandatory policies to limit marketing for or sales of foods and beverages that do not build health in the school environment. Such policies were only found in 28% of countries. Restrictions on competitive food sales were more common than limits on marketing (89% compared with 56% of existing policies), and only 12% of countries have policies to limit both marketing and competitive food sales in some capacity. Adoption of these policies could be limited by schools’ reliance on sales of competitive foods for revenue or marketing agreements with food companies that benefit the school via fundraising, sponsorships, or donations. The higher adoption frequency for competitive food restrictions may result from a prevailing belief that food marketing is not actually harmful or from concern that the industry could fight restrictions on marketing on the basis of free speech/expression rights, whereas it is easier—and there is more precedent—to simply put limitations on food vendors.

Notably, no national policies to limit marketing or competitive food sales in schools were found in LICs; the majority of policies were found in HICs, and vending machine bans were only found in HICs. This could be due to differences in food provision or availability in schools (for example, the presence of a cafeteria or canteen) or differences in school policy priorities or resources. In LICs, there may be a greater focus on encouraging children to attend school and consuming sufficient calories rather than a focus on restricting specific foods. However, given the growing double burden of under- and overnutrition in many LICs [34], these policy interventions could be important to consider for preempting worsening childhood obesity prevalence in the future. Further research is therefore needed on the school food environment in LICs and how the policy priorities may differ from countries of higher-income levels as this disparity in policies between LICs and wealthier nations.

The ways in which restricted foods and beverages are defined in policies are critical and vary widely, with some restricting competitive sales or marketing for entire categories of foods and drinks and others regulating based on a nutrient profiling model. The use of categorical lists may allow too much flexibility in the way schools or officials define these categories (for example, banning confectioneries but permitting baked goods that can have just as much sugar, salt, or saturated fat). Policies using a nutrient profiling model to identify products high in nutrients or ingredients of concern could lead to greater removal of nutritionally poor foods, insofar as any product exceeding a defined threshold would be regulated, regardless of category. This review found that sodium, fat (total or saturated), and sugar (total or added) were the most commonly restricted nutrients among the marketing and competitive food policies applying nutrient profiles to either specific food categories or to all food or beverage products. Still, profiling based on nutrient content may fail to capture certain products that are ultraprocessed or have relatively high content of saturated fat, sugar, or salt that fall just below regulatory thresholds [35].

Although multiple countries restricted foods or categories that were “processed,” this review found only 1 country, El Salvador, that explicitly included the term “ultraprocessed” in its list of restricted items in the country’s 2017 policy (Normativa de Tiendas y Cafetines Escolares Saludables) [36]. The global lack of restrictions on “ultraprocessed” products may result from discrepancies in how “ultraprocessed” is defined or the challenges of banning this wide category that includes shelf-stable and convenient food for school cafeterias. It could also be due to policies being implemented before the concept and potential health harms of ultraprocessing became more mainstream. Given children’s increasing intake of ultraprocessed foods worldwide [4,[37], [38], [39], [40], [41], [42], [43]] and mounting observational, epidemiological evidence of their association with numerous negative health risks [44], the lack of explicit limitations on ultraprocessed foods in the school environment represents a significant area for future improvement in policy design. Several countries (for example, Mexico, Brazil, and the United States) have integrated or are considering including avoidance of ultraprocessed foods into their national dietary guidelines, which could lead to more restrictions of ultraprocessed product availability in schools that align their food provision with dietary guidelines [[45], [46], [47]].

Policy impact is ultimately determined by effective implementation and adherence. Therefore, another area of interest was to assess the monitoring and evaluation guidelines available in the policy documentation. (This review did not attempt to document evidence of actual policy monitoring or enforcement activity, just the presence of these measures in policy language.) Interestingly, monitoring language was more commonly found in LMIC policy documents compared with UMIC and HIC. This pattern may reflect the more recent development of policies in LMICs and their use of updated policy guidance, suggesting that policies in UMICs and HICs should be revised to include more robust monitoring and enforcement guidelines.

At the same time, it is important to note that despite the inclusion of monitoring language, existing policy evaluations suggest that many countries do not appear to actively engage in sufficient monitoring activities. Even in countries with policies that more closely align with WHO recommendations by regulating both competitive foods and marketing [13], research shows that monitoring and enforcement are lacking. For example, India adopted a policy that includes specific restrictions on marketing and the sale of certain foods in schools based on their nutrient content, targeting foods high in sodium, saturated fat, *trans* fat, and sugar. This policy applies to areas within 50 meters of the school gate and applies to all types of educational institutions, ranging from preprimary to secondary ages. Despite this policy’s strength on article, an evaluation in India found low enforcement and continued availability of restricted food and beverage products within schools [48]. Similar findings were observed in Ecuador, where prohibited food products continue to be sold and consumed in schools despite regulations [49]. The lack of compliance with school food policies further emphasizes the need for monitoring and enforcement mechanisms alongside policy adoption. Policies that are ostensibly implemented but lack follow-through accomplish little more than the absence of a policy altogether. Including more explicit monitoring and enforcement mechanisms is a key area for improvement to strengthen future policy development.

The regions of Latin America & Caribbean, the Middle East & North Africa, and Europe & Central Asia had the highest number of countries with policies. Many countries in Latin America & Caribbean and Europe and Central Asia had both restrictive competitive food and marketing policies. Countries in Latin America & Caribbean, in particular, have been developing and implementing innovative school food environment policies to address rising obesity rates [[50], [51], [52]]. Having a strong regional presence of policies can allow for collaboration and increase pressure on neighboring nations to adopt similar policies.

On the other hand, few policies were found in Sub-Saharan Africa and South Asia. Most policies or national school feeding programs that did exist, particularly in sub-Saharan Africa, were focused on providing sufficient calories through school meals rather than regulating competitive foods or marketing. Implementing the latter could be especially important in these regions, where many countries are experiencing a double burden of malnutrition and must address undernourishment and concurrent rising obesity prevalence [34]. Limiting schoolchildren’s exposure to and consumption of micronutrient-poor, calorie-dense snack foods and drinks can prevent displaced consumption of more nourishing foods while encouraging better lifelong dietary preferences.

This review found that very few policies extended to areas immediately surrounding schools. Convenience stores and fast-food outlets clustered near schools provide students with easy access to relatively cheap, unhealthy foods, and often prominently display attention-grabbing advertising for these products [[53], [54], [55], [56]]. Policies should ideally restrict marketing and unhealthy food sales within close proximity to schools [1,13,57], but this review found only 7 countries had policies that did so, 2 of which had policies regulating both marketing and competitive foods in proximity to schools. Evidence from the United States, Finland, and Mexico shows that the foods to which children are exposed in close proximity to their schools can influence body composition [[58], [59], [60]]. Evidence also suggests that marketing around schools contributes to health disparities: Studies in New Zealand, Australia, Mexico, and the United States have found a higher concentration of outdoor advertising for unhealthy foods around schools serving lower-income or marginalized populations, relative to higher-income areas [56,[61], [62], [63]]. Considering the potential impact of marketing and ultraprocessed food availability in the areas around schools, this appears to be an underutilized mechanism for reducing inequity and improving the quality of children’s food environments.

### Strengths and limitations

This review had several strengths. First, including all United Nations -recognized countries provides a global snapshot of the current school food policy landscape. Given the heterogeneity of policy designs and challenges in interpretation, double coding a subset of countries to ensure inter-rater reliability between the 2 primary coders as well as frequent consultation with the research team on any unclear policy elements strengthened the study methods and improved the accuracy of results. Challenges in translation and finding primary policy documentation were addressed by confirming policy details with in-country experts, when necessary. Finally, including policy features such as nutrient profiling, the inclusion of areas around schools, and monitoring and enforcement mechanisms provides a more holistic picture of the school food policy landscape and gaps or strengths in current regulations that could impact efficacy. These aspects of the review enhance and aggregate policy data beyond what currently exists in databases such as the WHO’s GINA database, which researchers found to be a useful repository for basic policy information and links to documentation but did not always provide the most up-to-date policy information or complete policy details required for this study’s dataset.

This review also had several limitations. Although an extensive search was done to source primary policy documents for all regulations included in this review, it is possible that some were missed due to lack of availability online or language barriers. Accurate interpretation of policies may have been impacted due to translation errors or ambiguous policy language, despite attempts to prevent or mitigate such errors. Interpretation of the mandatory nature of guidelines may have also been impacted by translation errors or interpretation by gray or peer-reviewed literature. Finally, policies may have been modified or enacted after the data collection period. Policies were coded based on their status at the time of data collection. As such, this review does not capture the evolution of policies over time, changes in policies’ operationalization, or new policy implementation.

This review also focused only on national-level, mandatory policies and thus did not capture policies implemented by states, provinces, or more local jurisdictions. Policies emerged during this review that were relatively comprehensive in coverage and/or found to be effective in evaluations but were not mandatory or implemented at a national-level. For example, Australia, Canada, and the United Kingdom do not regulate the school food environment at the national-level, but policies at state, province, or territory levels are reportedly well-implemented and, in effect, achieve national coverage [[64], [65], [66], [67], [68], [69]]. In addition, some countries have comprehensive policies that are structured as voluntary guidelines yet have high reported compliance, such as in Finland and Japan [[70], [71], [72], [73], [74]]. These seem to be exceptions to the norm, as evidence shows voluntary policies are generally an ineffective approach to shaping and protecting a healthy food environment [75]. This review describes the global scope of these policies at the national-level. Although sub-national or regional reviews exist in some geographies [69], future reviews could explore a more comprehensive global policy analysis of sub-regional policies and/or the scope of these policies within sub-national or regional contexts.

Regarding competitive food sales, this review did not capture policies that preempt sales of less-healthy foods by restricting their procurement by schools and other public institutions, as in Brazil’s innovative school feeding program [76,77]. Future research should examine the global landscape for procurement policies, nudging interventions to promote healthy food behavior, pricing policies in schools, and policies implemented by sub-national jurisdictions and/or voluntarily [13].

In conclusion, Schools can be an ideal setting for forming and supporting healthy eating habits early in life, yet relatively few countries have national-level policies leveraging 2 key interventions shown to influence the school food environment: limiting unhealthy competitive food sales and food marketing in and around schools. Only 54 (28%) of the 193 countries in the world were found to have mandatory, national policies regulating either marketing for or sales of food and beverages that do not build health in the school environment, and only 24 countries (12%) had both policy types, indicating a need for increased policy adoption worldwide. This represents a large gap and opportunity for more countries to pursue policies that will improve and protect the school environment for all children. These policies are particularly important in low- and middle-income countries, where ultraprocessed foods are not yet fully established as a dominant component of the diet, and particularly for LICs, where there were no existing marketing or competitive food policies [78]. Future research should describe the global prevalence of policies aimed at improving other key aspects of the school food environment, particularly nutritional standards for school meals.

## Author contributions

The authors’ responsibilities were as follows – BMP, EAB: conceptualized the study; MP, EAB, LST, FRDC: drafted the codebook; GCC, KM: conducted data collection, coding, and policy analysis; MP: led data analysis and writing of the manuscript; all authors contributed to the writing and editing of the manuscript; and all authors: read and approved the final manuscript.

## Conflict of interest

BP, MP, EB, FDC, GC, KM, and LST report that financial support for this work was provided by Bloomberg Philanthropies. BP reports a relationship with the National Institutes of Health that includes funding grants. B P and FDC report relationships with the World Bank that include consulting or advisory. BP reports a relationship with Resolve to Saves Lives that includes consulting or advisory. LST reports a relationship with the National Institutes of Health that includes funding grants. LST reports a relationship with Resolve to Saves Lives that includes consulting or advisory. LST reports a relationship with Global Alliance in Nutrition that includes consulting or advisory. The authors declare no other known competing financial interests or personal relationships that could have appeared to influence the work reported in this article.

## Funding

We thank Bloomberg Philanthropies for financial support. The supporting source had no such involvement or restrictions regarding publication.

## Data availability

Data described in the manuscript, code book, and analytic code will be made available upon request.

## Footnotes

Appendix A Supplementary data to this article can be found online at https://doi.org/10.1016/j.advnut.2024.100254.
