# Supplementary material for: National Policies to Limit Food Marketing and Competitive Food Sales in Schools: A Global Scoping Review
Source: Adv Nutr. 2024 Jun 12;15(8):100254. doi: 10.1016/j.advnut.2024.100254 (PMC11295572; doi:10.1016/j.advnut.2024.100254)
Supplement: Multimedia component 1 [file mmc1.pdf]

National policies to limit food marketing and competitive food sales in schools: a global scoping review, Michelle Perry

Supplemental Materials

Supplemental Figure 1. Search Strategy **2**

Supplemental Table 1. Policy descriptions **3**

Appendix 1. Countries included in study sample (n=193),
World Bank region and income classifications, and policy status.........**17**

Appendix 2. Codebook **23**

Supplemental Figure 1. Search Strategy

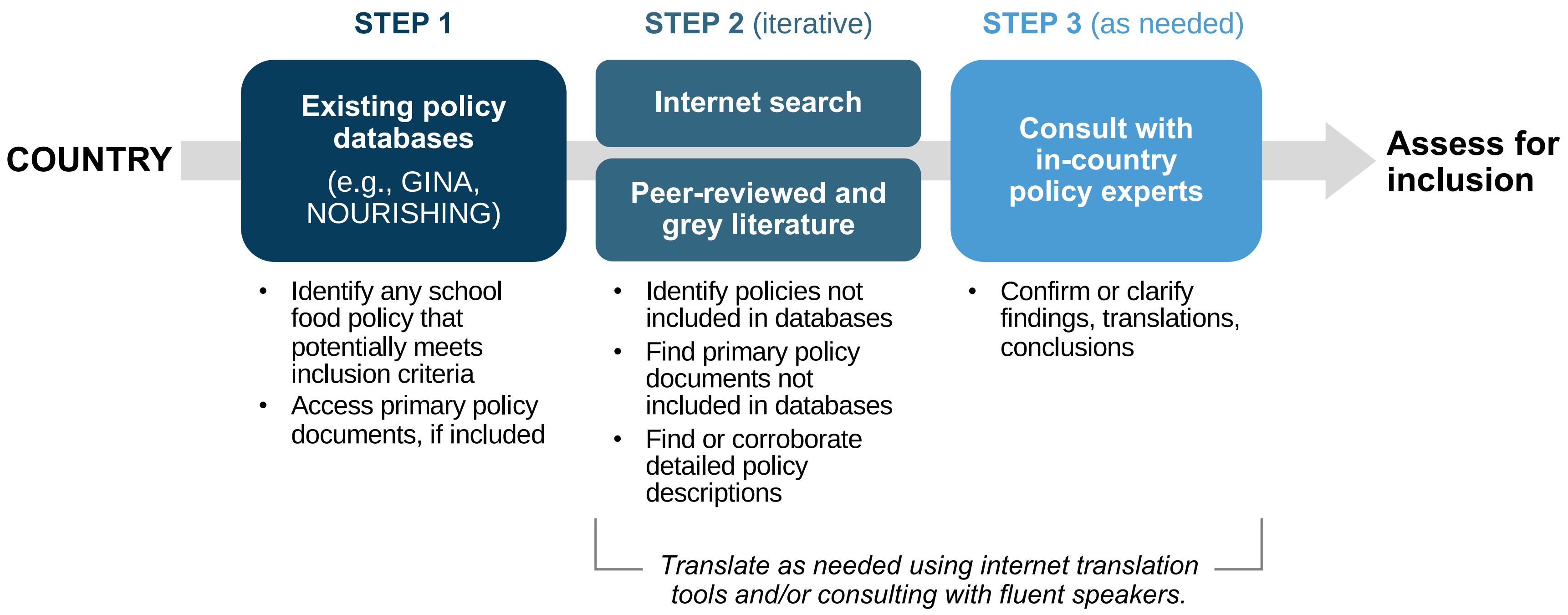

Supplemental Table 1. Policy descriptions

Country	Marketing policies	Competitive food policies
**Argentina**	According to Law 27642, *Ley de etiquetado frontal,* Article 12: Foods and beverages that contain at least one (1) warning label may not be offered, marketed, advertised, promoted or sponsored in educational establishments. This applies to pre-school, primary, and secondary levels of education within the National Educational System. Those who do not comply with the provisions of this law face sanctions, which can range from a warning and fines to the closure of the establishment. More information can be found in Title IV, Chapter III of Decree 274/2019l.	According to Law 27642, *Ley de etiquetado frontal,* Article 12: Foods and beverages that contain at least one (1) warning label may not be offered, marketed, advertised, promoted or sponsored in educational establishments. This applies to pre-school, primary, and secondary levels of education within the National Educational System. Those who do not comply with the provisions of this law face sanctions, which can range from a warning and fines to the closure of the establishment. More information can be found in Title IV, Chapter III of Decree 274/2019l.
**Bahamas**		*The Compulsory Standards for Healthy Lunch Meals in Bahamian Schools* prohibits the sale of Foods of Minimal Nutritional Value (FMNV), defined through a list of restricted food and beverage items. These restrictions apply to all "MOE schools, i.e. preschool, kindergarten, primary and secondary schools and apply to all free and paid lunches." The MOE is responsible for ensuring that vendors are in compliance with the policy.
**Bahrain**		*The Food Canteen List for the academic year 2016-2017*, which is interpreted as valid for current school years, details a list of food and beverages prohibited from being sold in the school cafeteria, such as chips, energy drinks and soft drinks, and candy, among others. Secondary sources confirm that this policy applies to all public elementary and secondary schools.
**Barbados**	*The Barbados School Nutrition Policy* prohibits food and beverage marketing of any kind for all pre-school and school children aged 3-17 years old. Food and beverage marketing activities are prohibited in or around all schools. These activities include direct advertising (e.g. advertisements placed inside or outside school buildings or on perimeter fencing or walls, billboards or distribution of promotional materials etc.); indirect advertising (e.g. corporate sponsored events, competitions, equipment, sportswear etc.); product sales; and market research (e.g. surveys and taste tests). METVT is responsible for implementation adherence with support from the MOHW. On-site compliance will be conducted by members of national coordinating committees. The framework for evaluation is being developed; there is no enforcement language at this time.	*The Barbados School Nutrition Policy* regulates the nutrient standards for foods and beverages served or sold in schools to all pre-school and school children aged 3-17 years old. This policy includes categorical restrictions (e.g. candy and soda) and nutrient/ingredient regulations on sweeteners, total calories, added sugar, total fat, saturated fat, trans fat, and sodium. The policy notes that vendors selling food and beverages cannot be within 300 feet of the property line of schools. METVT is responsible for implementation adherence with support from the MOHW. On-site compliance will be conducted by members of national coordinating committees. The framework for evaluation is being developed; there is no enforcement language at this time.

Country	Marketing policies	Competitive food policies
**Brunei Darussalam**		*The Panduan Penjualan Minuman Kantin Sekolah Negara Brunei Darussalam* (*Guidelines for the sale of beverages at the school canteen*) restricts the sale of foods included in a list, such as sports drinks, energy drinks, gum, and chips, among others. The restrictions apply to "all administrators, canteen entrepreneurs and food controllers who make the provision and sale of food and drinks in the school canteen." The MOE is responsible for monitoring the implementation of these guidelines.
**Bulgaria**		*Ordinance No. 37 of July 21, 2009* on healthy nutrition for students regulates the provision and sale of food and beverages in all institutions providing food to students. Specific food categories are restricted (e.g. no confectionery or pastries high in fat and sugar) and there is a list of allowable beverages. There are no thresholds for nutrient restrictions. There is no monitoring or enforcement language for the policy.
**Cabo Verde**	Decree-Law No. 11/2016 prohibits advertising or distribution of products with low nutritional value in all establishments of teaching in the national education system. The marketing regulations include defined categories, such as fried foods, candies, chewing gum, sausages, coffee, soft drinks, and more. They also regulate total fat and sodium. The key difference between the marketing and competitive food requirements is that the competitive food restrictions apply to the surrounding area (200 meters around school grounds), but the marketing restrictions do not. The policy is monitored every six months by the CONASE government body. Fines are in place for those who violate this policy.	*Decree-Law No. 11/2016* prohibits advertising or distribution of products with low nutritional value in all establishments of teaching in the national education system. The marketing regulations include defined categories, such as fried foods, candies, chewing gum, sausages, coffee, soft drinks, and more. They also regulate total fat and sodium. The key difference between the marketing and competitive food requirements is that the competitive food restrictions apply to the surrounding area (200 meters around school grounds), but the marketing restrictions do not. The policy is monitored every six months by the CONASE government body. Fines are in place for those who violate this policy.
**Chile**	According to *The Law on Nutritional Composition of Food and its Advertising* (Ley 20.606), "High in" products cannot be sold or advertised in schools. These include those with added sugar, added sodium, or added saturated fat that exceed set nutrient thresholds per 100 g (of food) or 100 mL (of beverage). Thresholds were implemented in phases with increasing stringency; as of 2019: 275 kcal/100 g of product (70 kcal/100 mL for beverages), 400 mg of sodium/100 g (100 mg/100 mL), 10 g of total sugar/100 g (5 g/100 mL), or 4 g of saturated fat/100 g (3 g/100 mL). The Chilean policy is unique in that all products that have marketing restrictions must also carry front-of-package warning labels (e.g., "high in sugar"). Monitoring and enforcement mechanisms are in place.	According to *The Law on Nutritional Composition of Food and its Advertising* (Ley 20.606), "High in" products cannot be sold or advertised in schools. These include those with added sugar, added sodium, or added saturated fat that exceed set nutrient thresholds per 100 g (of food) or 100 mL (of beverage). Thresholds were implemented in phases with increasing stringency; as of 2019: 275 kcal/100 g of product (70 kcal/100 mL for beverages), 400 mg of sodium/100 g (100 mg/100 mL), 10 g of total sugar/100 g (5 g/100 mL), or 4 g of saturated fat/100 g (3 g/100 mL). The Chilean policy is unique in that all products that have marketing restrictions must also carry front-of-package warning labels (e.g., "high in sugar"). Monitoring and enforcement mechanisms are in place.

Country	Marketing policies	Competitive food policies
**Costa Rica**	*Article 10 of Executive Decree No. 36910-MEP-S* (*Reglamento para el funcionamiento y administración del servicio de soda en los centros educativos públicos*) states that the Educational or Administrative Board must ensure compliance with the provisions of article 9 of this Regulation, and that in the cafeteria and the rest of the educational establishment no commercial advertising is used, placed, or displayed that directly or indirectly promotes the consumption of food products. This Regulation applies to all public preschool, primary and secondary educational centers or institutions within the national territory.	*Article 15 of Executive Decree No. 36910-MEP-S* prohibits the sale of specific foods and beverages in all soda services or food outlets installed in public preschool, primary and secondary educational centers. Certain foods are also restricted based on nutrient thresholds (e.g., bread for sandwiches cannot contain more than 20 grams of sugar or its equivalent in sweetener for every 100 grams of bread). The Board of Education or Administration and the Health and Nutrition Committee are responsible for monitoring adherence. Failure to comply could result in the dismissal of a member of the institution.
**Czechia**	*Decree No. 282/2016 Coll* regulates the requirements for foods for which advertising is permissible and which can be offered for sale and sold in schools and school facilities. The provisions have a list of allowable foods that can be sold and/or advertised in school settings. The decree also lists maximum permissible values for salt, fat, and sugar contained in all products. This law applies to all education levels below high school. Monitoring and enforcement mechanisms are in place.	*Decree No. 282/2016 Coll* regulates the requirements for foods for which advertising is permissible and which can be offered for sale and sold in schools and school facilities. The provisions have a list of allowable foods that can be sold and/or advertised in school settings. The decree also lists maximum permissible values for salt, fat, and sugar contained in all products. This law applies to all education levels below high school.
**Ecuador**	Acuerdo No. 0004-10 (*Reglamento Sustitutivo Para El Funcionamiento De Bares Escolares Del Sistema Nacional De Educación*) regulates the advertising, sale, and provision of foods in all school cafeterias. This policy prohibits food and drinks containing caffeine or artificial sweeteners and implements nutrient thresholds for all other foods. A National Committee monitors provincial committees that are responsible for implementing school meal regulations. The provincial health directorates also periodically monitor the operation of school food provision. Article 50 notes that noncompliance with the prohibitions contained in these regulations will be penalized in accordance with the provisions contained in the Organic Health Law, Consumer Defense Organic Law, its regulations, and other applicable regulations.	Acuerdo No. 0004-10 (*Reglamento Sustitutivo Para El Funcionamiento De Bares Escolares Del Sistema Nacional De Educación*) regulates the advertising, sale, and provision of foods in all school cafeterias. This policy prohibits food and drinks containing caffeine or artificial sweeteners and implements nutrient thresholds for all other foods. A National Committee monitors provincial committees that are responsible for implementing school meal regulations. The provincial health directorates also periodically monitor the operation of school food provision. Article. 50 notes that violations committed by food and beverage service providers in schools and noncompliance with the prohibitions contained in these regulations will be penalized in accordance with the provisions contained in the Organic Health Law, Consumer Defense Organic Law, its regulations, and other applicable regulations.
**El Salvador**	*Diario oficial el 16 de junio de 2017: Acuerdo N° 15-0733* regulates the marketing, sale, and provision of specific categories of processed and ultra-processed foods and any foods surpassing set levels of fat, sodium and sugar content. This regulation applies to all educational centers throughout the country. It is the responsibility of the School Board and the Ministry of Health to ensure that regulation requirements are adhered to. Any violations will be penalized in accordance with the provisions of the Consumer Protection Law.	*Diario oficial el 16 de junio de 2017: Acuerdo N° 15-0733* regulates the marketing, sale, and provision of specific categories of processed and ultra-processed foods and foods with specific levels of fat, sodium and sugar content. This regulation applies to all educational centers throughout the country. It is the responsibility of the School Board and the Ministry of Health to ensure that regulation requirements are adhered to. Any violations will be penalized in accordance with the provisions of the Consumer Protection Law.

Country	Marketing policies	Competitive food policies
**Estonia**	*The Advertising Act* notes that advertising is prohibited on the premises of pre-school childcare institutions, basic schools, upper secondary schools, and vocational educational institutions. Chapter 5 of the policy notes the requirements of states for the supervision of advertising; violations are punishable with a fine.	
**Fiji**		*The School Health Policy* enforces the implementation of the *School Canteen Guidelines*. These guidelines restrict specific food categories, such as sweetened drinks, confectionary, and chips, among others. The School Health Policy defines grade levels for the provision of School Canteen Guidelines as: "all Early Childhood Education Centres, Special, Primary and Secondary schools and Vocational Centres registered with MoEHA." The Canteen Committee is responsible for ensuring compliance with the Canteen Guidelines and National Food and Nutrition Policy for Schools. Enforcement mechanisms include fines and imprisonment.
**France**		*Article 30 of Law No. 2004-806 of August 9, 2004* relating to public health policy notes that vending machines for paid drinks and food products accessible to students are prohibited in schools from September 1, 2005. Secondary sources confirm this applies to primary and middle schools.
**Greece**		*Law Y1c/ G.P./oik 96605* (*School Package Hygiene Rules - Determination Of Available Products*) regulates the sale and provision of food in schools through a short list of specific categorical bans, such as sugar-sweetened beverages, and nutrient restrictions for certain types of foods. The regulations for school canteens apply within public and private primary and secondary schools. Monitoring checks are carried out regularly by designated bodies. Enforcement includes sanctions for noncompliance, issued through the National and Community Legislation.
**Hungary**	*Act XLVIII of 2008* (4) states that no advertisement of any kind may be disseminated in child welfare and child protection institutions, kindergartens, grammar schools, or in dormitories for students of grammar schools.	Competitive foods must meet requirements of healthy eating laid out in *Decree 20/2012 on the Operation of Public Education Institutions and the Use of Names of Public Education Institutions*. Foods and beverages may not be sold on school premises or at school events if they fall under the items taxed by the *Public Health Product Tax,* which includes specific categorical and nutrient restrictions. This policy applies to all grades from the first year of elementary school to the last year of secondary school studies. No monitoring or enforcement language is included in the policy.

Country	Marketing policies	Competitive food policies
**India**	*The Food Safety and Standards (Safe food and balanced diets for children in school) Regulations* of 2020 state that "No person shall advertise or market or sell or offer for sale including free sale, or permit sale of, food products high in saturated fat or trans-fat or added sugar or sodium in school campus or to school children in an area within fifty meters from the school gate in any direction." This regulation applies to all types of pre-primary, primary, elementary, and secondary schools. The State Food Authority is responsible for assessing compliance with the regulation.	*Food Safety and Standards (Safe food and balanced diets for children in school) Regulations* of 2020 state that "No person shall advertise or market or sell or offer for sale including free sale, or permit sale of, food products high in saturated fat or trans-fat or added sugar or sodium in school campus or to school children in an area within fifty meters from the school gate in any direction." This regulation applies to all types of pre-primary, primary, elementary, and secondary schools. The State Food Authority is responsible for monitoring compliance with these regulations.
**Iran**	Any form(s) of marketing in kindergartens, schools, and spaces specified for children have been banned. This is covered by *The Regulation on the Establishment and Monitoring of the Work and Activity of Advertising Centers* (1980) and *The Policies and Regulations Governing on Environmental Advertising* (2010). A working group composed of various public and private stakeholders monitors implementation.	
**Israel**		According to *The Supervision Of The Quality Of Food And Correct Nutrition In Educational Institutions Regulations*, 5779 – 2018, a food vendor in an educational institution may not sell "forbidden foods," including highly processed foods (e.g. hot dogs, pastries, candy bars), soft drinks, and energy drinks. *The Nutritional Marketing Regulations* also set standards for sodium, total sugar, and saturated fat allowed in food in schools. This policy applies to students aged 6-10, pulled from the related policy *Regulations For Supervision Of The Quality Of The Food And Correct Nutrition In After School Programs*, 5777 – 2017. While there is no monitoring language in the policy, enforcement mechanisms are included (fines for selling prohibited food.)
**Jamaica**	The *National School Nutrition Standards* published in August 2021 regulate marketing, competitive food sales, and school meal provision. The standards apply to all public early childhood Institutions, primary, and secondary institutions, and include a 200m perimeter around the school. Marketing efforts which should be prohibited in the school environment include: 1. Promotion of brands associated with HFSS products; 2. Sponsorship (by brands associated with HFSS products; and 3. Philanthropic activities tied to brands associated with HFSS products. The policy does not include any monitoring or enforcement language, but "monitoring and evaluation of the NSNP will be guided by a Monitoring and Evaluation Framework to be developed. "	The *National School Nutrition Standards* published in August 2021 regulate marketing, competitive food sales, and school meal provision. The standards apply to all public early childhood Institutions, primary and secondary institutions, including both within the school and a perimeter of 200m around the school. Marketing efforts which should be prohibited in the school environment include: 1. Promotion of brands associated with products which are high in salt, fat or sugar; 2. Sponsorship (by brands associated with products which are high in salt, fat or sugar); and 3. Philanthropic activities tied to brands associated with products which are high in salt, fat or sugar. The policy does not include any monitoring or enforcement language. It notes that, "Monitoring and evaluation of the NSNP will be guided by a Monitoring and Evaluation Framework to be developed. "

Country	Marketing policies	Competitive food policies
**Jordan**		The *Health requirements for school canteens and foods allowed and prohibited to be sold for the year 2012* lists out specific categories of foods that are prohibited from being sold in canteens, including drinks containing sugar, soft drinks, candy, chips, biscuits, and other types of foods. “Schools” was interpreted as all grades. There are no monitoring requirements for this policy; enforcement mechanisms include sanctions contained in the *Public Health Law*.
**Kiribati**	The *2014 Food Regulation and Standards* states "No person may use, arrange for, permit or authorise the advertising of any designated product or of any designated product trademark, on any school premises, at any childcare facility or at any health care facility." The policy notes that this applies to "school premises," which has been interpreted as all schools through secondary sources. This policy also applies to the surrounding area, as "no person may display, permit, authorise, or arrange for the display of any advertisement for a designated product within 200 metres of the entrance to a school or childcare facility or other place where children are likely to congregate." There are no monitoring requirements for the advertising policy; enforcement language includes fines and potential imprisonment for noncompliance.	
**Kuwait**		The mandatory standards for food in school, issued as a memo from the government, restricts "junk food" and fast food that is high in fat, calories, and low in nutrients. No specific nutrient thresholds were identified for this requirement. The policy applies to all schools in Kuwait. No monitoring or enforcement language is included.
**Latvia**	The *Energy Drinks Circulation Law* notes that the "advertising of energy drinks is prohibited in educational institutions and on the buildings and structures of these institutions." This law applies to all educational institutions. No monitoring or enforcement language was included in this law.	Under the *Hygiene requirements for general basic education, general secondary education and professional education institution*, foodstuffs and drinks that are high in sugar, salt, artificial colorings and flavorings are banned from kindergartens, primary, and secondary schools. They also ban the sale of caffeinated beverages/energy drinks and chewing gum (also included in the *Energy Drinks Circulation Law*). Schools also regulate the amount of sodium in competitive foods and beverages. The regulation does not include any monitoring or enforcement language.
**Lithuania**	A 2011 policy, *Description of the procedure for the organization of meals in pre-school education, general education schools and children's social care institutions*, states that food products that are banned from being served in school meals also "cannot be advertised in schools;" this includes: chips, sweets, items with sodium more than 0.4 g/100 g, carbonated drinks, energy drinks or coffee drinks, and food supplements. The policy contains a list of	According to the 2011 *Description of the procedure for the organization of meals in pre-school education, general education schools and children's social care institutions,* in schools where there is "free choice of snacks," they must be provided according to point 17 of the Procedure description, which bans: chips, sweets, items with sodium more than 0.4 g/100 g, carbonated drinks, energy drinks or coffee drinks, and food supplements. No monitoring or enforcement language is included in this policy.

Country	Marketing policies	Competitive food policies
	banned food additives, including sweeteners. No monitoring or enforcement language is included in this policy.	
**Malaysia**		Under the Circular of the Secretary General of the Ministry of Housing and Local Government No. 4/2012 *Guidelines for the Enforcement of the Prohibition of the Sale of Food and Beverages Outside School Fences by Local Authorities*, specific types of processed or junk foods (e.g. french fries, chips, chocolate, energy drinks, and carbonated beverages) are prohibited to be sold within 40 meters of schools. There is a complete ban on drinks that contain artificial sweeteners. The policy applies to all schools that fall under the Malaysian MOE." The KPKT (Ministry of Local Government) is responsible for monitoring implementation; no enforcement language is included.
**Malta**	Per the *Procurement of Food For Schools Regulations* (S.L.550.01 of 2018), schools shall not permit any advertising of or accept sponsorships by food products not allowed in accordance with the Advisory Council's criteria. These criteria categorically restrict HFSS items (e.g. fried foods, chips, energy drinks, and chocolates) and set nutrient/ingredient limits on total sugar, total fat, trans fat, saturated fat, sodium, caffeine, and non-nutritive sweeteners. This applies to all school grade levels. The Superintendent of Public Health is responsible for monitoring and enforcement of the policy. Fines are to be issued for those who violate the policy, with an increased fine for repeat offenses.	Per the *Procurement Of Food For Schools Regulations* (S.L.550.01 of 2018), only food that meets the requirements as outlined in the criteria issued by the Advisory Council may be offered for sale in schools. These foods include HFSS foods and those with certain limits of total sugar, total fat, trans fat, saturated fat, sodium, caffeine, and non-nutritive sweeteners. Some of the categorical bans include fried foods, chips, energy drinks, and chocolates. among others. This policy applies to primary and secondary schools. **The policy also includes a full ban on vending machines for the same grade levels.** The Superintendent of Public Health is responsible for monitoring and enforcement of the policy. Fines are issued for those who violate the policy.
**Mauritius**		*According to the Food (Sale of Food on Premises of Educational Institutions) Regulations 2009*, all food except those specified in a schedule are banned. Allowed foods include fruit, maize, nuts, yams, and other foods. The policy defines educational institutions as "any pre-school, primary school, secondary school, or pre-vocational school." No monitoring or enforcement language exists under the Sale of Food policy. However, this policy sits under the Food Act, which has penalties for those who do not follow the regulations, including fines and/or imprisonment. The regulation on the quantity of saturated fat included in competitive foods is included in the *Food Regulations made under the Food Act 1998.*

Country	Marketing policies	Competitive food policies
**Mexico**		According to DOF 16/05/2014, *Acuerdo Mediante El Cual Se Establecen Los Lineamientos Generales Para El Expendio Y Distribución De Alimentos Y Bebidas Preparados Y Procesados En Las Escuelas Del Sistema Educativo Nacional*, the preparation, sale and distribution of food and beverages in the schools of the National Educational System, that do not meet the nutritional criteria are prohibited. The requirements regulate the quantity of added sugar, total fat, trans fat, saturated fat, sodium, caffeine, and non-nutritive sweeteners. This policy applies to all basic, middle, and secondary school levels. Requirements are also in place for pre-schools. *The* School Consumption Establishments Committee is responsible for monitoring the quality and type of products that can be sold and distributed. Enforcement for noncompliance is included in article 75, sections IX and XIII of the *General Education Law*.
**Mongolia**	Per the policy *About Food Production and Service of Secondary Education Schools*, it is forbidden to advertise or sell "food prohibited for sale to children" in the school environment. This policy applies to all food production and services for children studying in general education schools and living in boarding houses. Prohibited items include carbonated drinks, enery drinks, chips, and ice cream, as well as any over set limits for salt, total sugar, added sugar, saturated fat, and trans-fat. The policy assigns monitoring responsibilities to the Control-Analysis and Evaluation Department; no enforcement language is included. A 2019 policy included a geographic requirement for shops within 150 meters of schools, but this was repealed in the 2021 amendment of the law and there is no longer a geographic requirement.	Per the policy About Food Production and Service of Secondary Education Schools, it is forbidden to advertise or sell "food prohibited for sale to children" in the school environment. This policy applies to all food production and services for children studying in general education schools and living in boarding houses. Prohibited items include carbonated drinks, enery drinks, chips, and ice cream, as well as any over set limits for salt, total sugar, added sugar, saturated fat, and trans-fat. The policy assigns monitoring responsibilities to the Control-Analysis and Evaluation Department; no enforcement language is included. A 2019 policy included a geographic requirement for shops within 150 meters of schools, but this was repealed in the 2021 amendment of the law and there is no longer a geographic requirement.
**Norway**	Per *The Act relating to Primary and Secondary Education and Training* (the Education Act), school owners must ensure that the pupils are not exposed to advertising that may exert commercial pressure, or that may substantially influence attitudes, behavior, and values, on the school premises, grounds, in textbooks, or other teaching resources. Thus, all advertising is banned in schools. The Ministry may issue further regulations. The Education Act applies to primary, lower secondary, and upper secondary education and training in publicly maintained schools and training establishments unless otherwise decided. The Education Act as a whole includes monitoring language (not specific to advertising); no enforcement mechanism is mentioned: "The County Governor supervises the municipal and county authorities' fulfilment of the duties bestowed upon them in or pursuant to Chapters 1-16 of the Education Act (municipal duties)."	

Country	Marketing policies	Competitive food policies
**Oman**		Two policies in Oman regulate competitive foods in schools: 1) The *School Hygiene Requirements* states that soft drinks, sports drinks, candy, and milk/yogurt with artificial flavors are not allowed in schools. 2) The *Guide to Health requirements for school cooperative societies/school canteens* bans other categories of foods and beverages (e.g. juices and drinks with less than 30% real fruit juice, potato chips of all kinds, fried potatoes, croissants and donuts). These policies apply to all educational institutions. Monitoring is conducted by a group of teachers, students, training staff, and nutritionists from the school health unit, inspectors, and food control agencies. In addition, the school health department nutritionist conducts unannounced inspections.
**Panama**	Food marketing is regulated by "*Guía Básica para la Oferta de Alimentos Saludables en Kioscos y Cafeterías de Centros Educativos.*" This document was made into law by Resolucion no. 49 in Panama. The regulations prohibit advertising, promotion, and sponsorship of food and beverages in educational institutions. Article 2 in resolution 49 notes that the policy applies to all primary and secondary schools. At the time of data collection, the policy stated that MOH will develop monitoring and surveillance measures in partnership with the MOE and the Authority for Consumer Protection and Defense of Competition.	Competitive foods are regulated by "Guía Básica para la Oferta de Alimentos Saludables en Kioscos y Cafeterías de Centros Educativos," made into law by Resolucion no 49 in Panama. The policy prohibits the sale of carbonated beverages, unhealthy snacks, and other HFSS, as well as prohibiting all non-nutritive sweeteners and setting nutrient thresholds for calories, total sugar, total fat, and sodium. Article 2 in resolution 49 notes that the policy applies to all primary and secondary schools. The policy notes that "Surveillance and control of all aspects of public health in school kiosks and cafeterias will be the responsibility of the local health authority and its health team." There is a monitoring checklist included as an appendix, as well as a list of responsibilities of MOH. No enforcement language is included.
**Peru**		Law No. 30021 (*Law to promote healthy eating for children and adolescents)* bans foods with octagonal labels (i.e. high in sodium, sugar, saturated fats, trans fats) and foods and beverages exceeding established limits for sodium, added sugar, saturated fat and trans-fat from being sold in schools. Further, *Ministerial Resolution No. 195-2019/MINSA* restricts any food and beverages that contain non-nutritive sweeteners. The policy applies to all educational institutions. The MOE coordinates monitoring of healthy kiosks, canteens, and cafeterias. No enforcement language is included.

Country	Marketing policies	Competitive food policies
**Philippines**	According to *Policy and Guidelines on Healthy Food and Beverage Choices in Schools and in DepEd Offices*, the "marketing of red category foods and beverages shall not be allowed in schools." The policy notes that it applies to all public primary and secondary schools. Red foods are both defined through specific nutrient limits (saturated fat, trans fat, added sugar, and sodium) and categorical restrictions (e.g. soft drinks, ice cream, chocolate, and hard candies.) Caffeine is also banned. The regional and division monitors are expected to monitor compliance with these guidelines quarterly and monthly. Any personnel who violate any provision of these guidelines faces repercussions per the DepEd Order No 49, the Revised Rules of Procedure of the Department of Education on Administrative Cases.	According to *Policy and Guidelines on Healthy Food and Beverage Choices in Schools and in DepEd Offices*, "Canteens in schools shall not sell foods and beverages high in fat and or sugar and or sodium as listed in table 6," which are "red foods". Red foods are both defined through specific nutrient limits (saturated fat, trans fat, added sugar, and sodium) and categorical restrictions (e.g. soft drinks, ice cream, chocolate, and hard candies.) Caffeine is also banned. The policy notes that it applies to all public primary and secondary schools. Regional/divisional monitors are responsible for monitoring compliance with these guidelines. Anyone who violates the guidelines faces sanctions under DepEd *Order No 49, the Revised Rules of Procedure of the Department of Education on Administrative Cases*.
**Poland**	*Regulation of the Minister of Health of July 26, 2016 on foodstuff for sale to children and adolescents in schools* includes a list of approved food groups that allows only foods within those groups that meet nutritional standards to be allowed to be marketed or sold on school grounds. This act regulates the marketing and sale of these foods in all schools, including pre-school and kindergartens. The policy also restricts food above a specified threshold of added sugar, total sugar, total fat, and sodium. Non-nutritive sweeteners are also regulated. *The Original Act* establishes a monitoring council and fines up to PLN 5,000 for noncompliance.	*Regulation of the Minister of Health of July 26, 2016 on foodstuff for sale to children and adolescents in schools* includes a list of approved food groups that allows only foods within those groups that meet nutritional standards to be allowed to be marketed or sold on school grounds. This act regulates the marketing and sale of these foods in all schools, including pre-school and kindergartens. The policy also restricts food above a specified threshold of added sugar, total sugar, total fat, and sodium. Non-nutritive sweeteners are also regulated. *The Original Act* establishes a monitoring council and fines up to PLN 5,000 for noncompliance.
**Portugal**	*Law 30/2019* amended the country's 14th Advertising Code and introduced restrictions on advertising directed to children (<16 years) of any HFSS food/ beverages. The Law took effect October 2019 and specifically prohibits advertisement of HFSS foods in pre-schools through secondary education establishments, sports, cultural, and recreational activities organized by these schools, in public playgrounds, and within a radius of 100 meters of these spaces. There are fines for non-compliance.	Different from the marketing policy, "*Despacho n.º 8127/2021, de 17 de agosto*" regulates "products harmful to health in vending machines available in schools and the rules for school buffets, including information regarding the foods that may or may not be available." In school buffets and vending machines, specific categories of food and beverages are banned, including deli meat, sweets, chips, and soft drinks, among others. Portugal has lists of foods to be promoted and to be limited: vending machines can only sell items on the promoted list.
**Qatar**		*The Guidance for supervisors of school canteens for the academic year 2018-2019* regulates categories and nutrient thresholds for foods and beverages sold in school (e.g. energy drinks, sweet drinks, candies, chips). The policy also includes nutrient limits (calories, total fat, saturated fat, trans fat, added sugar, total sugar, and sodium) and bans caffeine; NNS is limited for milk products. The policy references only primary and secondary schools for certain food groups. The Environmental Health Department/Ministry of Public Health are responsible for monitoring violations; there is a list of fines for violations.

Country	Marketing policies	Competitive food policies
**Republic of Korea**		On May 8, 2009, KFDA announced that of the foods listed in Annex 1 of the *The Special Act on the Safety Management of Children's Dietary Life*, foods that are high in calories and low in nutrients will be banned from sales. Foods within this category are subject to the nutrient thresholds on calories, total sugar, saturated fat, and sodium. The term "school" means elementary schools, middle schools, high schools, and special schools under Article 2 of the *Elementary and Secondary Education Act*. The policy requires the establishment of a Children's Dietary Life Safety Management Committee to monitor compliance. Fines for noncompliance are included in the policy. As policy did not list the annex with specific food categories that are restricted, the list was pulled from the following: https://unicefeaproinasactoolkit.files.wordpress.com/2017/09/the-special-act-on-childrens-dietary-life-safety-management_seoul_korea-republic-of_5-15-2009.pdf
**Romania**		*Law no. 123/2008 for a healthy diet in pre-university education units* states that HFSS foods and all drinks except water are banned, with specific limits (per 100g) for sugar, salt, fat, and a per-unit limit for calories. The Ministry of Public Health also prepares and updates a list of foods not recommended for preschoolers and schoolchildren, according to the recommendations of nutrition specialists.
**San Marino**		Under the *National Guidelines for School Catering*, only secondary schools can have vending machines. There are no specific nutrient restrictions for what can be included in the vending machines. The Food, Hygiene, and Nutrition Service (SIAN) ensures compliance with regulations; no enforcement mechanisms are included.
**Saudi Arabia**		*The Regulations of Health Conditions for School Canteens* restrict the sale of specific categories of competitive foods, including energy drinks, doughnuts, sports drinks, meat, and fried foods, among others. The policy defines the school age group as those between 6-18 years, covering elementary, middle, and high school. There is no monitoring or enforcement language in the policy.
**Serbia**	According to the *Advertising Law (Policy - Zakon o Oglašavanju)*, "Advertising in a school, preschool institution or other institution intended for children or minors is not allowed, unless it serves to protect the general interests of children or minors, as well as educational, educational and sports activities." There are no nutritional regulations associated with this restriction. Monitoring is completed by the ministry responsible for trade affairs. Fines are in place for violations of this law.	

Country	Marketing policies	Competitive food policies
**Seychelles**	According to the *National School Nutrition Policy*, "there should be no active promotion or advertising of full fat crisps, fatty and sugary foods within the dining room." There are no further restrictions or regulations for the types of food restricted in the cafeteria. The policy does not explicitly detail which grades it applies to, but it was inferred that it applies to primary and secondary through the categories used within the list of required nutrient values. The policy notes that "to ensure that the policy is implemented successfully, schools will need to be regularly guided, monitored and evaluated through various multi-sectoral bodies which would include a National School Nutrition Committee and School Nutrition Action Groups at school level." There is no enforcement language included.	According to the *National School Nutrition Policy*, FLNV (e.g. lollies, candies, corn chips, and carbonated beverages) should not be offered to students on school premises during activities. The policy also bans NNS. There are no other nutrient/ingredient regulations in place. The policy does not explicitly detail which grades the policy applies to, but it was inferred that the policy requirements apply to "primary and secondary" through the categories used within the list of required nutrient values. The policy notes that "to ensure that the policy is implemented successfully, schools will need to be regularly guided, monitored and evaluated through various multi-sectoral bodies which would include a National School Nutrition Committee and School Nutrition Action Groups at school level." There is no enforcement language included
**Slovakia**	*Regulation No. 527/2007* of the Slovak Ministry of Health prohibits the marketing of caffeinated beverages in its requirements for facilities for children and young people. There are no monitoring or enforcement mechanisms included.	Regulation No. 527/2007 of the Slovak MOH prohibits the marketing of caffeinated beverages in its requirements for facilities for children and young people. Under same law, "the assortment of goods in buffets, vending machines and other forms of ambulatory sales set up within the facility for children and youth" cannot contain "beverages containing caffeine."
**Slovenia**		Under the *School Nutrition Act In Slovenia (ZŠolPre-1)*, a ban on installation of vending machines in primary and secondary school areas has been in force since May 2010. There are no nutrient regulations associated with this law. The law includes language on internal and external monitoring, internal and external control, and fines associated with non-compliance, specifically including vending machines.
**Spain**	According to Law 17/2011 of July 5 (*de seguridad alimentaria y nutrición*), "schools and nursery schools are declared as advertising-free spaces [...] Food promotion campaigns, nutritional education or promotion of sports or physical activity in schools [...] must be previously authorized by the competent educational authorities, in accordance with the criteria established by the authorities." The policy includes sanctions for non-compliance but no routine monitoring requirements.	*According to Law 17/2011 of July 5* (*de seguridad alimentaria y nutrición*), "In nursery schools and schools, the sale of foods and beverages with a high content of saturated fatty acids, trans fatty acids, salt and sugars will not be allowed. These contents will be established by regulation." The policy does not include nutrient thresholds. The policy includes a variety of sanctions for non-compliance but does not include monitoring language.
**Thailand**	According to the *Notification of the Ministry of Education Re: Measures and Approaches to Enhance Knowledge and Skills Related to Oral Health Care and Selection of Dental Services* (Announced on 11 June 2020), Thailand's MOE banned promotion activities for all foods and beverages in educational institutions. There are no nutrient thresholds associated with this policy. There is also no monitoring or enforcement language.	

Country	Marketing policies	Competitive food policies
**Trinidad and Tobago**		In May 2017, the Trinidad and Tobago MOH introduced a ban on the sale or serving of sugar-sweetened drinks at all government and government-assisted schools. The policy banned sweetened drinks, including soft drinks, juice drinks, flavoured water, sports/energy drinks, tea and coffee, and milk-based drinks with added sugars and artificial sweeteners. The policy does not include any monitoring or enforcement language.
**Ukraine**		According to MOH Resolution No. 305, *On the approval of norms and the Procedure for the feeding organization in educational institutions and children's health and recreation institutions,* all educational institutions in Ukraine have restrictions for foods available in schools. This includes categorical restrictions, such as carbonated drinks, energy drinks, and certain types of meats, among others, as well as nutrient/ingredient thresholds for non-nutritive sweeteners, caffeine, total sugar, added sugar, trans fat, and sodium. The policy does not include any monitoring or enforcement language.
**United States**	Under the *Healthy, Hunger-Free Kids Act of 2010*, the USDA restricts food and beverage marketing that does not meet the nutrient standards for Smart Snacks. The policy institutes marketing restrictions based on nutrient thresholds for competitive food sales, which includes calories, total sugar, total fat, saturated fat, trans fat, sodium, and caffeine. This policy applies to elementary, middle, and high schools. The final rule requires each district to assess compliance with its school wellness policy and make this assessment available to the public at least once per 3 years. There is no enforcement language included in the policy.	*The National School Lunch Program and School Breakfast Program: Nutrition Standards for All Foods Sold in School as Required by the Healthy, Hunger-Free Kids Act of 2010 (HHFKA)* establishes standards for foods sold in schools. The regulations include specific requirements for elementary, middle, and high schools (limits differ by category). Elementary and middle school foods and beverages must be caffeine free with the exception of naturally occurring trace amounts. Competitive food regulations include limiting foods that do not meet the following: "(1) meet all of the proposed competitive food nutrient standards; and (2) be a grain product that contains 50% or more whole grains by weight or have whole grains as the first ingredient*; or (3) have as the first ingredient* one of the non-grain main food groups: fruits, vegetables, dairy, or protein foods (meat, beans, poultry, seafood, eggs, nuts, seeds, etc.); or (4) be a combination food that contains at least ¼ cup fruit and/or vegetable." The products must also fall within the limits for calories, total sugar, total fat, saturated fat, trans fat, and sodium. Section 208 of the HHFKA requires state agencies to ensure local educational agencies comply with these requirements; any noncompliant school or school food authority may be fined.

Country	Marketing policies	Competitive food policies
**Uruguay**	According to Decreto N° 60/014, *Reglamentacion De La Ley 19.140 Relativo A La Proteccion De La Salud De La Poblacion Infantil Y Adolescente A Traves De La Promocion De Habitos Alimenticios Saludables*, advertising and sales are prohibited for products that do not meet the standards of the list of recommended food and beverage groups, including its display at sales outlets, for all educational institutions. The list of standards includes nutrient threshold for total calories, total sugar, added sugar, total fat, saturated fat, trans fat, sodium, and artificial sweeteners. There is no monitoring or enforcement language included.	According to Decreto N° 60/014, *Reglamentacion De La Ley 19.140 Relativo A La Proteccion De La Salud De La Poblacion Infantil Y Adolescente A Traves De La Promocion De Habitos Alimenticios Saludables*, the sale of products that do not meet the standards of the list of recommended food and beverage groups is prohibited for all educational institutions. The list of standards includes nutrient/ingredient thresholds on total calories, total sugar, total fat, saturated fat, trans fat, sodium, and artificial sweeteners. There is no monitoring or enforcement language included.
**Vanuatu**		According to the *Sweet Drinks Policy*, schools and kindergartens prohibit sweet drinks, defined as drinks which contain sugar, sweetener or flavouring and have low nutritional value (e.g. carbonated or 'fizzy' drinks, cordials, sports drinks, energy drinks, fruit drinks, fruit juices, sugarcane juice, flavoured milks, and drinks with added sugar.) The MOE, in partnership with each Provincial Education Office, Zone Curriculum Advisors, and School Improvement Officer, are responsible for monitoring compliance; no enforcement language is included.

* Note that some policy descriptions include translated language used to code the policies. Translations may not be completely accurate in word choice.
FMNV/FLNV: foods of minimal/low nutritional value
HFSS: food and beverage products high in saturated fat, salt and sugar
MOE: Ministry of Education; MOH: Ministry of Health

Appendix 1. Countries included in study sample (n=193), World Bank region and income classifications, and policy status

Country[1]	World Bank region	World Bank income classification	National, mandatory policy *restricting marketing and/or competitive food sales in schools*
**Afghanistan**	South Asia	Low Income	
**Albania**	Europe & Central Asia	Upper Middle Income	
**Algeria**	Middle East & North Africa	Lower Middle Income	
**Andorra**	Europe & Central Asia	High Income	
**Angola**	Sub-Saharan Africa	Lower Middle Income	
**Antigua and Barbuda**	Latin America & Caribbean	High Income	
**Argentina**	Latin America & Caribbean	Upper Middle Income	✓
**Armenia**	Europe & Central Asia	Upper Middle Income	
**Australia**	East Asia & Pacific	High Income	
**Austria**	Europe & Central Asia	High Income	
**Azerbaijan**	Europe & Central Asia	Upper Middle Income	
**Bahamas**	Latin America & Caribbean	High Income	✓
**Bahrain**	Middle East & North Africa	High Income	✓
**Bangladesh**	South Asia	Lower Middle Income	
**Barbados**	Latin America & Caribbean	High Income	✓
**Belarus**	Europe & Central Asia	Upper Middle Income	
**Belgium**	Europe & Central Asia	High Income	
**Belize**	Latin America & Caribbean	Upper Middle Income	
**Benin**	Sub-Saharan Africa	Lower Middle Income	
**Bhutan**	South Asia	Lower Middle Income	
**Bosnia and Herzegovina***	Europe & Central Asia	Upper Middle Income	
**Botswana**	Sub-Saharan Africa	Upper Middle Income	
**Brazil**	Latin America & Caribbean	Upper Middle Income	
**Brunei Darussalam**	East Asia & Pacific	High Income	✓
**Bulgaria***	Europe & Central Asia	Upper Middle Income	✓
**Burkina Faso**	Sub-Saharan Africa	Low Income	
**Burundi**	Sub-Saharan Africa	Low Income	
**Cabo Verde**	Sub-Saharan Africa	Lower Middle Income	✓
**Cambodia**	East Asia & Pacific	Lower Middle Income	
**Cameroon**	Sub-Saharan Africa	Lower Middle Income	
**Canada**	North America	High Income	

Country[1]	World Bank region	World Bank income classification	National, mandatory policy *restricting marketing and/or competitive food sales in schools*
**Central African Republic**	Sub-Saharan Africa	Low Income	
**Chad**	Sub-Saharan Africa	Low Income	
**Chile**	Latin America & Caribbean	High Income	✓
**China**	East Asia & Pacific	Upper Middle Income	
**Colombia**	Latin America & Caribbean	Upper Middle Income	
**Comoros**	Sub-Saharan Africa	Lower Middle Income	
**Congo**	Sub-Saharan Africa	Lower Middle Income	
**Costa Rica**	Latin America & Caribbean	Upper Middle Income	✓
**Côte d'Ivoire**	Sub-Saharan Africa	Lower Middle Income	
**Croatia**	Europe & Central Asia	High Income	
**Cuba***	Latin America & Caribbean	Upper Middle Income	
**Cyprus***	Europe & Central Asia	High Income	
**Czechia**	Europe & Central Asia	High Income	✓
**Democratic People's Republic of Korea**	East Asia & Pacific	Low Income	
**Democratic Republic of the Congo**	Sub-Saharan Africa	Low Income	
**Denmark**	Europe & Central Asia	High Income	
**Djibouti**	Middle East & North Africa	Lower Middle Income	
**Dominica**	Latin America & Caribbean	Upper Middle Income	
**Dominican Republic**	Latin America & Caribbean	Upper Middle Income	
**Ecuador**	Latin America & Caribbean	Upper Middle Income	✓
**Egypt**	Middle East & North Africa	Lower Middle Income	
**El Salvador**	Latin America & Caribbean	Lower Middle Income	✓
**Equatorial Guinea**	Sub-Saharan Africa	Upper Middle Income	
**Eritrea**	Sub-Saharan Africa	Low Income	
**Estonia**	Europe & Central Asia	High Income	✓
**Eswatini**	Sub-Saharan Africa	Lower Middle Income	
**Ethiopia**	Sub-Saharan Africa	Low Income	
**Federated States of Micronesia**	East Asia & Pacific	Lower Middle Income	
**Fiji**	East Asia & Pacific	Upper Middle Income	✓
**Finland***	Europe & Central Asia	High Income	
**France**	Europe & Central Asia	High Income	✓
**Gabon**	Sub-Saharan Africa	Upper Middle Income	
**Gambia**	Sub-Saharan Africa	Low Income	

**Country**[1]	**World Bank region**	**World Bank income classification**	**National, mandatory policy *restricting marketing and/or competitive food sales in schools***
**Georgia**	Europe & Central Asia	Upper Middle Income	
**Germany**	Europe & Central Asia	High Income	
**Ghana**	Sub-Saharan Africa	Lower Middle Income	
**Greece**	Europe & Central Asia	High Income	✓
**Grenada***	Latin America & Caribbean	Upper Middle Income	
**Guatemala**	Latin America & Caribbean	Upper Middle Income	
**Guinea**	Sub-Saharan Africa	Low Income	
**Guinea-Bissau**	Sub-Saharan Africa	Low Income	
**Guyana**	Latin America & Caribbean	Upper Middle Income	
**Haiti**	Latin America & Caribbean	Lower Middle Income	
**Honduras***	Latin America & Caribbean	Lower Middle Income	
**Hungary**	Europe & Central Asia	High Income	✓
**Iceland**	Europe & Central Asia	High Income	
**India**	South Asia	Lower Middle Income	✓
**Indonesia**	East Asia & Pacific	Lower Middle Income	
**Iraq**	Middle East & North Africa	Upper Middle Income	
**Ireland**	Europe & Central Asia	High Income	
**Islamic Republic of Iran**	Middle East & North Africa	Lower Middle Income	✓
**Israel***	Middle East & North Africa	High Income	✓
**Italy**	Europe & Central Asia	High Income	
**Jamaica***	Latin America & Caribbean	Upper Middle Income	✓
**Japan***	East Asia & Pacific	High Income	
**Jordan**	Middle East & North Africa	Upper Middle Income	✓
**Kazakhstan**	Europe & Central Asia	Upper Middle Income	
**Kenya**	Sub-Saharan Africa	Lower Middle Income	
**Kiribati**	East Asia & Pacific	Lower Middle Income	✓
**Kuwait***	Middle East & North Africa	High Income	✓
**Kyrgyzstan**	Europe & Central Asia	Lower Middle Income	
**Lao People's Democratic Republic**	East Asia & Pacific	Lower Middle Income	
**Latvia**	Europe & Central Asia	High Income	✓
**Lebanon**	Middle East & North Africa	Lower Middle Income	
**Lesotho**	Sub-Saharan Africa	Lower Middle Income	
**Liberia**	Sub-Saharan Africa	Low Income	
**Libya**	Middle East & North Africa	Upper Middle Income	
**Liechtenstein**	Europe & Central Asia	High Income	

Country[1]	World Bank region	World Bank income classification	National, mandatory policy *restricting marketing and/or competitive food sales in schools*
**Lithuania**	Europe & Central Asia	High Income	✓
**Luxembourg**	Europe & Central Asia	High Income	
**Madagascar**	Sub-Saharan Africa	Low Income	
**Malawi**	Sub-Saharan Africa	Low Income	
**Malaysia**	East Asia & Pacific	Upper Middle Income	✓
**Maldives**	South Asia	Upper Middle Income	
**Mali**	Sub-Saharan Africa	Low Income	
**Malta**	Middle East & North Africa	High Income	✓
**Marshall Islands**	East Asia & Pacific	Upper Middle Income	
**Mauritania**	Sub-Saharan Africa	Lower Middle Income	
**Mauritius**	Sub-Saharan Africa	Upper Middle Income	✓
**Mexico**	Latin America & Caribbean	Upper Middle Income	✓
**Monaco**	Europe & Central Asia	High Income	
**Mongolia**	East Asia & Pacific	Lower Middle Income	✓
**Montenegro**	Europe & Central Asia	Upper Middle Income	
**Morocco**	Middle East & North Africa	Lower Middle Income	
**Mozambique**	Sub-Saharan Africa	Low Income	
**Myanmar**	East Asia & Pacific	Lower Middle Income	
**Namibia**	Sub-Saharan Africa	Upper Middle Income	
**Nauru**	East Asia & Pacific	High Income	
**Nepal***	South Asia	Lower Middle Income	
**Netherlands**	Europe & Central Asia	High Income	
**New Zealand**	East Asia & Pacific	High Income	
**Nicaragua**	Latin America & Caribbean	Lower Middle Income	
**Niger**	Sub-Saharan Africa	Low Income	
**Nigeria**	Sub-Saharan Africa	Lower Middle Income	
**North Macedonia**	Europe & Central Asia	Upper Middle Income	
**Norway**	Europe & Central Asia	High Income	✓
**Oman***	Middle East & North Africa	High Income	✓
**Pakistan**	South Asia	Lower Middle Income	
**Palau***	East Asia & Pacific	Upper Middle Income	
**Panama**	Latin America & Caribbean	High Income	✓
**Papua New Guinea**	East Asia & Pacific	Lower Middle Income	
**Paraguay**	Latin America & Caribbean	Upper Middle Income	
**Peru**	Latin America & Caribbean	Upper Middle Income	✓
**Philippines**	East Asia & Pacific	Lower Middle Income	✓

Country[1]	World Bank region	World Bank income classification	National, mandatory policy *restricting marketing and/or competitive food sales in schools*
**Plurinational State of Bolivia**	Latin America & Caribbean	Lower Middle Income	
**Poland***	Europe & Central Asia	High Income	✓
**Portugal**	Europe & Central Asia	High Income	✓
**Qatar**	Middle East & North Africa	High Income	✓
**Republic of Korea***	East Asia & Pacific	High Income	✓
**Republic of Moldova***	Europe & Central Asia	Upper Middle Income	
**Romania**	Europe & Central Asia	High Income	✓
**Russian Federation**	Europe & Central Asia	Upper Middle Income	
**Rwanda**	Sub-Saharan Africa	Low Income	
**Saint Kitts and Nevis**	Latin America & Caribbean	High Income	
**Saint Lucia**	Latin America & Caribbean	Upper Middle Income	
**Saint Vincent and the Grenadines**	Latin America & Caribbean	Upper Middle Income	
**Samoa***	East Asia & Pacific	Lower Middle Income	
**San Marino**	Europe & Central Asia	High Income	✓
**São Tomé and Príncipe**	Sub-Saharan Africa	Lower Middle Income	
**Saudi Arabia**	Middle East & North Africa	High Income	✓
**Senegal**	Sub-Saharan Africa	Lower Middle Income	
**Serbia**	Europe & Central Asia	Upper Middle Income	✓
**Seychelles**	Sub-Saharan Africa	High Income	✓
**Sierra Leone**	Sub-Saharan Africa	Low Income	
**Singapore**	East Asia & Pacific	High Income	
**Slovakia**	Europe & Central Asia	High Income	✓
**Slovenia**	Europe & Central Asia	High Income	✓
**Solomon Islands**	East Asia & Pacific	Lower Middle Income	
**Somalia**	Sub-Saharan Africa	Low Income	
**South Africa**	Sub-Saharan Africa	Upper Middle Income	
**South Sudan**	Sub-Saharan Africa	Low Income	
**Spain**	Europe & Central Asia	High Income	✓
**Sri Lanka**	South Asia	Lower Middle Income	
**Sudan**	Sub-Saharan Africa	Low Income	
**Suriname**	Latin America & Caribbean	Upper Middle Income	
**Sweden**	Europe & Central Asia	High Income	
**Switzerland**	Europe & Central Asia	High Income	

**Country**[1]	**World Bank region**	**World Bank income classification**	**National, mandatory policy *restricting marketing and/or competitive food sales in schools***
**Syrian Arab Republic**	Middle East & North Africa	Low Income	
**Tajikistan**	Europe & Central Asia	Lower Middle Income	
**Thailand**	East Asia & Pacific	Upper Middle Income	✓
**Timor-Leste**	East Asia & Pacific	Lower Middle Income	
**Togo**	Sub-Saharan Africa	Low Income	
**Tonga***	East Asia & Pacific	Upper Middle Income	
**Trinidad and Tobago**	Latin America & Caribbean	High Income	✓
**Tunisia**	Middle East & North Africa	Lower Middle Income	
**Türkiye**	Europe & Central Asia	Upper Middle Income	
**Turkmenistan**	Europe & Central Asia	Upper Middle Income	
**Tuvalu**	East Asia & Pacific	Upper Middle Income	
**Uganda**	Sub-Saharan Africa	Low Income	
**Ukraine***	Europe & Central Asia	Lower Middle Income	✓
**United Arab Emirates**	Middle East & North Africa	High Income	
**United Kingdom**	Europe & Central Asia	High Income	
**United Republic of Tanzania**	Sub-Saharan Africa	Lower Middle Income	
**United States of America**	North America	High Income	✓
**Uruguay**	Latin America & Caribbean	High Income	✓
**Uzbekistan**	Europe & Central Asia	Lower Middle Income	
**Vanuatu**	East Asia & Pacific	Lower Middle Income	✓
**Venezuela**	Latin America & Caribbean	N/A	
**Viet Nam**	East Asia & Pacific	Lower Middle Income	
**Yemen**	Middle East & North Africa	Low Income	
**Zambia**	Sub-Saharan Africa	Low Income	
**Zimbabwe**	Sub-Saharan Africa	Lower Middle Income	

[1] Per 2022-2023 United Nations designations
* Query sent to in-country contacts for clarification on policy interpretation.

Appendix 2. Codebook

Variable #	Variable/ Field Name	Form Name	Section Header	Field Label	Field Type	Choices, Calculations, OR Slider Labels	Skip Logic
1	record_id	Regulations		Record ID<br><br>**Country Name*	text	[open field]	
2	data_entry	Regulations		Person performing country search/data input	dropdown	1, Kayla Mardin|2, Grace Chamberlin| 3, Emily Busey| 4, Michelle Perry	
3	data_dt	Regulations		Date of Data Capture	text(date_mdy)		
4	Country	Regulations		Name of country.	dropdown	*choose from list of countries	
5	reg_any	Regulations		Does this country have any <u>required</u> national **restrictions** that limit exposure or access to unhealthy foods or beverages in the school or surrounding environment (i.e., marketing restrictions, competitive food/beverage standards, school meal standards)?<br><br>**This does NOT include F/V subsidies or incentives, farm-to-school programs, school gardens, or voluntary guidelines.*	yesno	Yes, No	If no, skip to 'comment'
6	mkt_any	Marketing Regulations	General	Does this country have any national marketing restrictions in schools (i.e., complete ban, nutrition standards for marketing, timing or placement restrictions, etc.)?<br><br>*Food marketing includes oral, written, or graphic statements made for the purpose of promoting the sale of a food or beverage product. These include only regulations only on products that can or cannot be promoted. Any regulations on food that can or cannot be sold belongs in the 'Competitive Food Regulations'	yesno	Yes, No	If no, skip to 'Competitive Food Regulations' section
7	mkt_implement	Marketing Regulations	General	Are these regulations currently active?	yesno	Yes, No	

Variable #	Variable/ Field Name	Form Name	Section Header	Field Label	Field Type	Choices, Calculations, OR Slider Labels	Skip Logic
8	mkt_implement_notes	Marketing Regulations	General	If not currently active, please provide details.	notes	[open field]	
9	mkt_ban	Marketing Regulations	Complete Ban	Is there a complete ban on all food and beverage marketing on school campuses?<br><br>** A complete ban means absolutely no food and beverage marketing allowed on school grounds. If there are any specific details of what can or cannot be promoted, this constitutes a partial ban. If marketing of "healthy foods" is allowed, this would be a partial ban.*	yesno	Yes, No	If yes, go to next question. If no, skip to 'mkt_partial'
10	mkt_ban_age<br>**mkt_ban_age_1*<br>**mkt_ban_age_2*<br>**mkt_ban_age_3*<br>**mkt_ban_age__4*	Marketing Regulations	Complete Ban	If so, for which of the following school levels does the complete marketing ban apply?	Checkbox	1, Preschools|2, Primary schools (grades K-6)|3, Lower Secondary schools (grades 6-8)|4, Upper Secondary schools (grades 9-12)	If all are yes, skip to 'mkt_surround' If any are no, continue to 'mkt_partial'
11	mkt_ban_age_notes	Marketing Regulations	Complete Ban	Include the policy language indicating applicable age/grade range.	text	[open field]	
12	mkt_partial	Marketing Regulations	Partial Ban	Are there any partial bans on food and beverage marketing on school campuses?	yesno	Yes, No	If yes, go to next question. If no, skip to 'mkt_surround'
13	mkt_partial_age<br>**mkt_partial_age_1*<br>**mkt_partial_age_2*<br>**mkt_partial_age_3*<br>**mkt_partial_age___4*	Marketing Regulations	Partial Ban	If so, does the partial marketing ban apply to:	Checkbox	1, Preschools|2, Primary schools (grades K-6)|3, Lower Secondary schools (grades 6-8)|4, Upper Secondary schools (grades 9-12)	
14	mkt_partial_age_notes	Marketing Regulations	Partial Ban	Include the policy language indicating applicable age/grade range.	text	[open field]	

Variable #	Variable/ Field Name	Form Name	Section Header	Field Label	Field Type	Choices, Calculations, OR Slider Labels	Skip Logic
15	mkt_place	Marketing Regulations	Criteria	Do the marketing restrictions include placement criteria (i.e. placement within school)?	yesno	Yes, No	
16	mkt_cat	Marketing Regulations	Criteria	Do the marketing restrictions apply to specific categories of food (i.e., HFSS, UPFs- ultra processed foods, food groups- potato chips, cakes, sweets, etc.)?	yesno	Yes, No	If yes, go to next question. If no, continue to 'mkt_healthy'
17	mkt_cat_notes	Marketing Regulations	Criteria	If so, please specify categories subject to marketing restrictions.	notes	[open field]	
18	mkt_nutr	Marketing Regulations	Criteria	Do the marketing restrictions include nutritional standards for which products may be promoted?<br><br>**Includes calories, sugar, fat, sodium, caffeine, artificial sweeteners/NCS/NNS.*	yesno	Yes, No	If yes, go to next question. If no, skip to 'mkt_surround'
19	mkt_thresh<br>**mkt_thresh__1*<br>**mkt_thresh__2*<br>**mkt_thresh__3*<br>**mkt_thresh__4*<br>**mkt_thresh__5*<br>**mkt_thresh__6*<br>**mkt_thresh__7*<br>**mkt_thresh__8*<br>**mkt_thresh__9*	Marketing Regulations	Criteria	Please select all categories with thresholds/nutritional standards.	Checkbox	1, Total Calories | 2, Total Sugar| 3, Added Sugar|4, Total Fat|5, Saturated Fat|6, Trans Fat| 7, Sodium| 8, Caffeine| 9, Artificial Sweeteners/NCS/NNS|	
20	mkt_surround	Marketing Regulations	Surrounding Area	Does this country have any national marketing regulations for the area surrounding schools?<br>*(Regulations must explicitly state that they apply to the area surrounding schools, with or without specific distance ranges, in order to be counted here; rules generally applicable to the whole population/country do not apply)*	yesno	Yes, No	If yes, go to next question. If no, skip to 'mkt_monitor'

Variable #	Variable/ Field Name	Form Name	Section Header	Field Label	Field Type	Choices, Calculations, OR Slider Labels	Skip Logic
21	mkt_surround_age<br>*mkt_surround_age_1*<br>*mkt_surround_age_2*<br>*mkt_surround_age_3*<br>*mkt_surround_age_4*	Marketing Regulations	Surrounding Area	If so, to which grade levels do these regulations apply?	Checkbox	1, Preschools|2, Primary schools (grades K-6)|3, Lower Secondary schools (grades 6-8)|4, Upper Secondary schools (grades 9-12)	
22	mkt_surround_age_notes	Marketing Regulations	Surrounding Area	Include the policy language indicating applicable age/grade range.	text	[open field]	
23	mkt_monitor	Marketing Regulations	Monitoring & Enforcement	Is there any form of monitoring for these marketing regulations?	yesno	Yes, No	
24	mkt_enforce	Marketing Regulations	Monitoring & Enforcement	Is there any form of enforcement of these marketing regulations?<br><br>Note: "Enforcement" implies actual penalties/punitive action for not abiding by regulations contained with the law or policy. If this is not found, check "no."	yesno	Yes, No	
25	mkt_me_notes	Marketing Regulations	Monitoring & Enforcement	Please include any relevant information about monitoring or enforcement.	notes	[open field]	
26	comp_any	Competitive Food Regulations	General	Does this country have any national regulations restricting sales of competitive foods and/or beverages in schools (i.e., complete ban, vending restrictions, timing or placement restrictions, nutrition standards for competitive foods, etc.)?<br><br>**Competitive foods* are any foods or beverages sold in schools separate from the federally funded school meal programs. Includes canteens, tuck shops, vending, and vendors coming onto school grounds.*	yesno	Yes, No	If no, skip to 'School Meal Regulations' section
27	comp_implement	Competitive Food Regulations	General	Are these regulations currently active?	yesno	Yes, No	

Variable #	Variable/ Field Name	Form Name	Section Header	Field Label	Field Type	Choices, Calculations, OR Slider Labels	Skip Logic
28	comp_implement_notes	Competitive Food Regulations	General	If not currently active, please provide details.	notes	[open field]	
29	comp_ban	Competitive Food Regulations	Complete Ban	Do the competitive food restrictions include a complete ban on competitive foods in schools?<br><br>** A complete ban means absolutely no outside food and beverage marketing are allowed to be sold on school grounds, with the exception of food and beverages sold through the federally funded school meal program.*	yesno	Yes, No	If yes, go to next question. If no, skip to 'comp_partial'
30	comp_ban_age<br>**comp_ban_age_1*<br>**comp_ban_age_2*<br>**comp_ban_age_3*<br>**comp_ban_age___4*	Competitive Food Regulations	Complete Ban	If so, to which grade levels does the complete competitive food ban apply?	Checkbox	1, Preschools|2, Primary schools (grades K-6)|3, Lower Secondary schools (grades 6-8)|4, Upper Secondary schools (grades 9-12)	If all are yes, skip to 'comp_surround' If any are no, continue to ‘comp_partial'
31	comp_ban_age_notes	Competitive Food Regulations	Complete Ban	Include the policy language indicating applicable age/grade range.	text	[open field]	
32	comp_partial	Competitive Food Regulations	Partial Ban	Are there any partial bans on food and beverage ban on school campuses?	yesno	Yes, No	If yes, go to next question. If no, skip to 'vend_ban'
33	comp_partial_age<br>**comp_partial_age_1*<br>**comp_partial_age_2*<br>**comp_partial_age_3*	Competitive Food Regulations	Partial Ban	If so, to which grades does the partial competitive food ban apply?	Checkbox	1, Preschools|2, Primary schools (grades K-6)|3, Lower Secondary schools (grades 6-8)|4, Upper Secondary schools (grades 9-12)	
34	comp_partial_age_notes	Competitive Food Regulations	Partial Ban	Include the policy language indicating applicable age/grade range.	text	[open field]	

Variable #	Variable/ Field Name	Form Name	Section Header	Field Label	Field Type	Choices, Calculations, OR Slider Labels	Skip Logic
35	vend_ban	Competitive Food Regulations	Vending Restrictions	Do the competitive food restrictions include a total ban on vending machines in schools?	yesno	Yes, No	If yes, go to next question. If no, skip to 'comp_time'
36	vend_ban_age **vend_ban_age_1* **vend_ban_age_2* **vend_ban_age_3* **vend_ban_age__4*	Competitive Food Regulations	Vending Restrictions	If so, to which grades does the complete vending machine ban apply?	Checkbox	1, Preschools|2, Primary schools (grades K-6)|3, Lower Secondary schools (grades 6-8)|4, Upper Secondary schools (grades 9-12)	
37	vend_ban_age_notes	Competitive Food Regulations	Vending Restrictions	Include the policy language indicating applicable age/grade range.	text	[open field]	
38	comp_time	Competitive Food Regulations	Criteria	Do the competitive food regulations include timing criteria (i.e., what time of day these foods are available, time of day children can access vending machines, etc.)?	yesno	Yes, No	
39	comp_place	Competitive Food Regulations	Criteria	Do the competitive food restrictions include placement criteria (i.e. placement or location within school)?	yesno	Yes, No	
40	comp_cat	Competitive Food Regulations	Criteria	Do the competitive food restrictions limit specific categories of food (i.e., HFSS, UPFs- ultra processed foods, food groups- potato chips, cakes, sweets, etc.)?	yesno	Yes, No	If yes, go to next question. If no, continue to 'comp_caff'
41	comp_cat_notes	Competitive Food Regulations	Criteria	If so, please specify categories subject to competitive food restrictions.	notes	[open field]	
42	comp_caff	Competitive Food Regulations	Criteria	Are there regulations on caffeine in competitive beverages?	radio	1, Complete ban |2, Limits on quantity present in all beverages|3, Limits on quantity in specific beverage categories|4, No restrictions	[comp_caff]=3, show [comp_caff_spec]

Variable #	Variable/ Field Name	Form Name	Section Header	Field Label	Field Type	Choices, Calculations, OR Slider Labels	Skip Logic
43	comp_caff_spec			Please specify specific beverage categories subject to caffeine limits.	notes	[open field]	
44	comp_nns_food	Competitive Food Regulations	Criteria	Are there regulations on the use of artificial sweeteners/NCS/NNS in competitive **foods**?	radio	1, Complete ban |2, Limits on quantity present in all foods|3, Limits on quantity in specific food categories|4, No restrictions	[comp_nns_food] =3, show [comp_nns_food _spec]
45	comp_nns_food_spec	Competitive Food Regulations	Criteria	Please specify specific food categories subject to artificial sweeteners/NCS/NNS limits.	notes	[open field]	
46	comp_nns_bev	Competitive Food Regulations	Criteria	Are there regulations on the use of artificial sweeteners/NCS/NNS in competitive **beverages**?	yesno	Yes, No	[comp_nns_bev] =3, show [comp_nns_bev_ spec]
47	comp_nns_bev_spec	Competitive Food Regulations	Criteria	Please specify specific beverage categories subject to artificial sweeteners/NCS/NNS limits.	radio	1, Complete Ban on use of artificial sweeteners/NCS/N NS| 2, Limits on quantity of artificial sweeteners/NCS/N NS	
48	comp_food_nutr	Competitive Food Regulations	Criteria	Do the competitive food regulations include nutritional standards for **foods**?<br><br>**Includes calories, sugar, fat, sodium.*	yesno	Yes, No	If yes, go to next question. If no, continue to 'comp_bev_nutr'
49	comp_food_thresh<br>**comp_food_thresh__1*<br>**comp_food_thresh__2*<br>**comp_food_thresh__3*<br>**comp_food_thresh__4*<br>**comp_food_thresh__5*<br>**comp_food_thresh__6*<br>**comp_food_thresh__7*	Competitive Food Regulations	Criteria	Please select all categories with thresholds/nutritional standards.	Checkbox	1, Total Calories | 2, Total Sugar| 3, Added Sugar|4, Total Fat|5, Saturated Fat|6, Trans Fat| 7, Sodium	

Variable #	Variable/ Field Name	Form Name	Section Header	Field Label	Field Type	Choices, Calculations, OR Slider Labels	Skip Logic
50	comp_bev_nutr	Competitive Food Regulations	Criteria	Do the competitive food regulations include nutritional standards for **beverages**?<br><br>**Includes calories, sugar, fat, sodium.*	yesno	Yes, No	If yes, go to next question. If no, continue to 'comp_surround'
51	comp_bev_thresh<br>**comp_bev_thresh__1*<br>**comp_bev_thresh__2*<br>**comp_bev_thresh__3*<br>**comp_bev_thresh__4*<br>**comp_bev_thresh__5*<br>**comp_bev_thresh__6*<br>**comp_bev_thresh__7*	Competitive Food Regulations	Criteria	Please select all categories with thresholds/nutritional standards.	Checkbox	1, Total Calories | 2, Total Sugar| 3, Added Sugar|4, Total Fat|5, Saturated Fat|6, Trans Fat| 7, Sodium	
52	comp_surround	Competitive Food Regulations	Surrounding Area	Does this country have any competitive food regulations for the area surrounding schools?	yesno	Yes, No	If yes, go to next question. If no, skip to 'comp_monitor'
53	comp_surround_age<br>**comp_surround_age_1*<br>**comp_surround_age_2*<br>**comp_surround_age_3*<br>**comp_surround_age_4*	Competitive Food Regulations	Surrounding Area	If so, to which grades do these regulations apply?	Checkbox	1, Preschools|2, Primary schools (grades K-6)|3, Lower Secondary schools (grades 6-8)|4, Upper Secondary schools (grades 9-12)	
54	comp_surround_age_notes	Competitive Food Regulations	Surrounding Area	Include the policy language indicating applicable age/grade range.	text	[open field]	
55	comp_monitor	Competitive Food Regulations	Monitoring & Enforcement	Is there any form of monitoring for these competitive food regulations?	yesno	Yes, No	
56	comp_enforce	Competitive Food Regulations	Monitoring & Enforcement	Is there any form of enforcement of these marketing regulations?<br>*Note: "Enforcement" implies actual penalties/punitive action for not abiding by regulations contained with the law or policy. If this is not found, check "no."*	yesno	Yes, No	

Variable #	Variable/ Field Name	Form Name	Section Header	Field Label	Field Type	Choices, Calculations, OR Slider Labels	Skip Logic
57	comp_me_notes	Competitive Food Regulations	Monitoring & Enforcement	Please include any relevant information about monitoring or enforcement.	notes	[open field]	
58–102	*These codes were used to review national mandatory regulations on categories, nutrients, or ingredients served in school meals. These results will be reported and discussed in a separate, forthcoming manuscript by the same authors.*						
103	comment	General Comments		[NOT REQUIRED]<br>Include any general notes here:<br><br>*This includes: Any ambiguity or uncertainty / missing information; Barriers in the search process; Regional laws that cover most of the country without an official national policy; Whether a country is considering starting or stopping a policy; Any unique policies that are worth highlighting in the paper	notes	[open field]	
104	sources	General Comments		Include hyperlinks for the main sources used to find the information in this form.	notes	[open field]	
105	Zotero	General Comments		Have you entered these sources in Zotero?	radio	1, Yes | 2, Not yet	
